# Eco-Friendly Conformal and Self-Adhesive Electrochemical Sensors for Sweat Monitoring

**DOI:** 10.1021/acsami.5c07032

**Published:** 2025-09-16

**Authors:** Xiaohe Wang, Muling Zeng, Mabel Torrens, Pengfei Niu, César Fernández Sánchez, Martí Gich, Anna Roig

**Affiliations:** † State Key Laboratory of Precision Measuring Technology & Instruments, Tianjin University, Tianjin 300072, China; ‡ Institut de Ciència de Materials de Barcelona, ICMAB (CSIC), Campus UAB, 08193 Bellaterra, Spain; § Instituto de Microelectrónica de Barcelona, IMB-CNM (CSIC), Campus UAB, 08193 Bellaterra, Spain; ∥ Centro de Investigación Biomédica en Red de Bioingeniería, Biomateriales y Nanomedicina (CIBER-BBN), 28029 Madrid, Spain

**Keywords:** wearables, bacterial cellulose, sustainability, sweat sensor, electrochemical sensors, alcohol detection

## Abstract

Wearable sweat-sensing platforms represent a transformative advancement in noninvasive, real-time health monitoring, enabling personalized healthcare. For in vivo applications, sensor substrate materials require biocompatibility, secure adhesion, and, preferably, environmental sustainability. However, existing substrate materials fail to meet some of those requirements. This study introduces bacterial cellulose (BC) as a novel sensor substrate, leveraging its printability, biocompatibility, self-adhesion, and eco-friendliness. A wearable sweat sensor was fabricated by screen-printing conductive inks onto BC films. A key challenge addressed was the hydrophilicity of BC, which can cause liquid penetration and disrupt signal stability. To solve this, an approach was developed where the electrical tracks are sandwiched between two hydrophobic layers to fully avoid liquid interference and ensure stable electrochemical performance. The sensor was further functionalized with the alcohol oxidase enzyme to enable reliable alcohol detection in sweat at the relevant concentration range. This work demonstrates the feasibility of BC-based sensors for their application in wearable health monitoring, meanwhile promoting sustainable technological innovations in personalized healthcare and well-being technologies.

## Graphical abstract

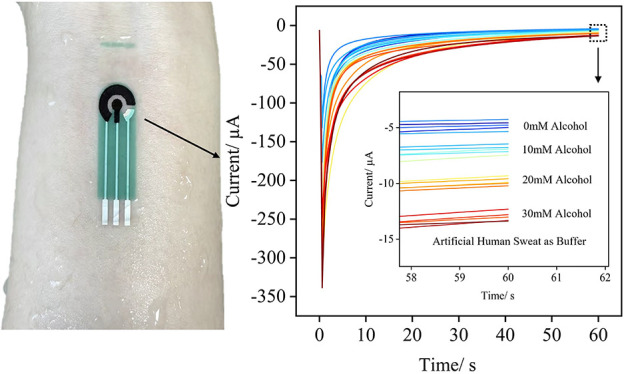

## 1 Introduction

Recent advancements in technology and the growing attention to personal health have created an increasing demand for efficient, user-friendly health monitoring solutions.^1^ Traditional health assessments, while reliable, are often hindered by their dependence on specialized medical personnel, complex equipment, long processing times, and high costs, limiting their suitability for continuous, real-time health monitoring in daily life.^2^ In this context, wearable sensors have emerged as transformative analytical devices that are compact, flexible, and capable of real-time physiological monitoring, offering immediate feedback to users.^3^ Among these, wearable sweat sensors have gained particular attention for their capability to provide noninvasive and continuous monitoring of human health by analyzing sweat.^4^ Sweat contains a diverse array of biomarkers, including electrolytes,^5,6^ metabolites,^6–8^ hormones,^9^ proteins,^10,11^ amino acids,^12,13^ and vitamins,^14^ making it a valuable diagnostic medium for detecting various diseases. Electrochemical sensing, known for its high sensitivity and selectivity,^15–17^ has become the cornerstone of wearable sweat sensors,^5,6,8–14,18,19^ enabling precise biomarker analysis and the improvement of health monitoring and disease diagnostics.

Currently, research on wearable sweat electrochemical sensors primarily focuses on sensor surface modification and systematic integration with back-end circuits.^20,21^ Advanced sweat detection platforms have been developed to measure a wide range of biomarkers in sweat.^22,23^ Traditional wearable sensors, however, often rely on synthetic petrol-based polymer substrates, which are nondegradable, raising concerns about user discomfort and ecological contamination. The most commonly used substrate materials for wearable electrochemical sensors include polyethylene terephthalate (PET),^10,24^ polyimide (PI) films,^12,23,25^ or polydimethylsiloxane (PDMS).^26,27^ While these materials offer flexibility, they are inherently dense and hydrophobic, necessitating the use of an additional adhesive layer to ensure firm contact with the skin.^10–12,23–27^ This attachment method not only compromises user comfort but also raises concerns regarding material biocompatibility and breathability. Prolonged skin adherence can lead to irritation, manifesting as redness, swelling, or allergic reactions,^28^ thereby limiting the long-term usability of such sensors. In addition, prolonged use of such impermeable adhesive materials can cause sweat accumulation between the skin and sensor, thus weakening the attachment of adhesive layers and presenting challenges for the sensor’s wearability and signal stability.^29^ More importantly, the plastic substrate of these wearable sensors, especially those designed for single use, produces large amounts of plastic waste in the environment, posing a serious threat to our ecosystem.^30^ Just as an example, the increased use of nonbiodegradable plastic waste from photonic components at their end-of-life could lead to the accumulation of up to 12,000 million tons in landfills or natural environments by 2050, particularly in developing nations.^31^ These issues highlight the critical need for innovative solutions that combine wearing stability, enhanced user comfort, and eco-friendliness.

Recent progress in green materials science has introduced porous biomaterials derived mainly from natural polymers (e.g., cellulose,^32–35^ chitosan hydrogel,^36^ starch-based hydrogel,^37^ silk fibroin^38^) and few synthesized materials like poly­(lactic acid)^39^ as promising candidates for wearable devices due to their excellent biodegradability, biocompatibility and low environmental impact. These materials not only reduce pollution from plastic but also enhance skin compatibility—a critical factor for long-term wearability. Here, the potential of bacterial cellulose (BC) is explored as an alternative sensor substrate. As a naturally biosynthesized polymer produced by various bacteria such as *Komagataeibacter* and *Pseudomonas*,^40^ BC stands out for being porous, mechanically robust, biodegradable, biocompatible^41–43^ and presenting a self-adhesive behavior.^44^ It can securely adhere to human skin through its inherent hydrophilicity, eliminating the need for additional adhesives. Consequently, the manufacturing process is simplified, facilitating green manufacturing techniques for wearable devices. Simultaneously, based on our advanced manufacturing techniques, an ultrathin and porous BC film is produced and exhibits excellent printability by screen-printing, unprecedented breathability, efficient thermal conductivity, and vertical water transfer, significantly enhancing user comfort during extended wear. The biocompatibility of BC further guarantees safe and irritation-free skin contact. Furthermore, its biodegradability is essential to ensure environmental sustainability, as it undergoes complete degradation in natural settings within a few months, preventing ecological harm upon disposal after use.

In this study, a wearable sweat electrochemical sensor was fabricated by screen-printing on BC flat films used as a substrate, designed to align with the principles of sustainable chemistry and green engineering. However, the intrinsic superhydrophilicity of BC poses challenges, mainly related to uncontrolled solution penetration, leading to high capacitive currents and unstable analytical performance, which has not been resolved. To address these issues, we developed a sandwich-structured sensor design, in which the electrical tracks are printed between two hydrophobic layers so that they are fully isolated from the environment. This architecture ensures that just the area of the electrodes gets in contact with the sample and guarantees a stable and reproducible electrochemical performance. To test the device applicability for sweat analysis, the electrode surface was modified with the alcohol oxidase enzyme to produce a BC sensor that could be applied to the reliable detection of alcohol in sweat ranging from 0 to 30 mM, consistent with physiological levels in the human body.^45,46^ The legal threshold for driving is defined as a blood alcohol concentration (BAC) of ≥0.08% in America (equivalent to 17.3 mM in sweat), ≥0.03% in Japan (equivalent to 6.5 mM in sweat), and ≥0.05% in most European countries (equivalent to 10.8 mM in sweat).^47^ The sensitivity and minimum detection concentration of the presented alcohol sensor were adjusted to fulfill the requirements for identifying illegal drunk driving behavior. The successful detection of alcohol in sweat underscores the practical applicability of our BC sensor and highlights its promising potential for the precise monitoring of other low-concentration biomarkers and analytes in this biofluid.

## 2 Experimental Section

### 2.1 Reagents and Materials

Potassium nitrate (≥99%), alcohol oxidase solution (AOx solution, ref#A2404, 10–40 units/mg protein, *Pichia pastoris*), Nafion (ref#117, ∼5%), glutaraldehyde (50% in H_2_O), bovine serum albumin (BSA, ≥98%), ethanol (≥99.5%), agarose (≥99.5%), dextrose (≥99%), citric acid (≥99.7%), Na_2_HPO_4_·12H_2_O (≥99%), peptone (≥99%) and yeast extract (≥99%) were purchased from Sigma-Aldrich (Spain). Phosphate buffered saline (PBS, 10×, PH 7.4, RNase free, ≥99.7%) was purchased from Thermofisher Scientific (Spain). Artificial human sweat was purchased from Biochemazone (ref#BZ320, USA). *Komagataeibacter xylinus* strain was purchased from Colección Española de Cultivos Tipo (Spain). All the solutions were prepared in deionized (DI) water. Prussian Blue (PB) carbon ink (ref#C2070424P2), Ag/AgCl ink (ref#C2130809D5) and dielectric ink (ref#D2070423P5) were obtained from SunChemical (Spain). 0.5 mm-thick PET sheets (Autostat WP20) were purchased from MacDermid (UK). 0.05 mm-thick PI film was purchased from Texiang (China). All reagents were used as received without further purification.

### 2.2 Fabrication of BC Film on a PET Substrate

To prepare BC films, Hestrin–Schramm (HS) culture medium was formulated by dissolving 5 g of dextrose, 8 g of peptone, 4 g of yeast extract, 12 g of Na_2_HPO_4_·12H_2_O, and 2 g of citric acid in 1 L of DI water. The medium was subsequently sterilized at 120 °C. After the sterilizaton process, the HS medium was inoculated with the liquid culture, where the proportion of HS medium to the liquid inoculum was always (v/v) 9:1. Thus, the bacterial inoculum comprised 10% of the total volume (Figure 1A­(1)). The mixture was then incubated at 30 °C for 7 days in the dark. Collected BC pellicles (Figure 1A­(2)) were first immersed in ethanol 50% for 10 min before being moved to boiling water for 10 min, twice, and then, the films were stirred in a hot 0.1 M NaOH solution for 10 min twice. Finally, the films were washed with DI water to restore the neutral pH (Figure 1A­(3)). The cleaned as-obtained pellicles of BC (12 × 12 cm^2^) were subsequently sandwiched between two PET sheets and, while maintained under a load of 2 kg (Figure 1A­(4)), were dried under vacuum until fully desiccated (Figure 1A­(5)) to obtain BC films. Finally, the upper PET sheet was carefully peeled away, yielding a dry and flat BC film adhered to the PET substrate (Figure 1A­(6)). PET substrates were cleaned with pure ethanol and air-dried before use.

**Figure 1:**
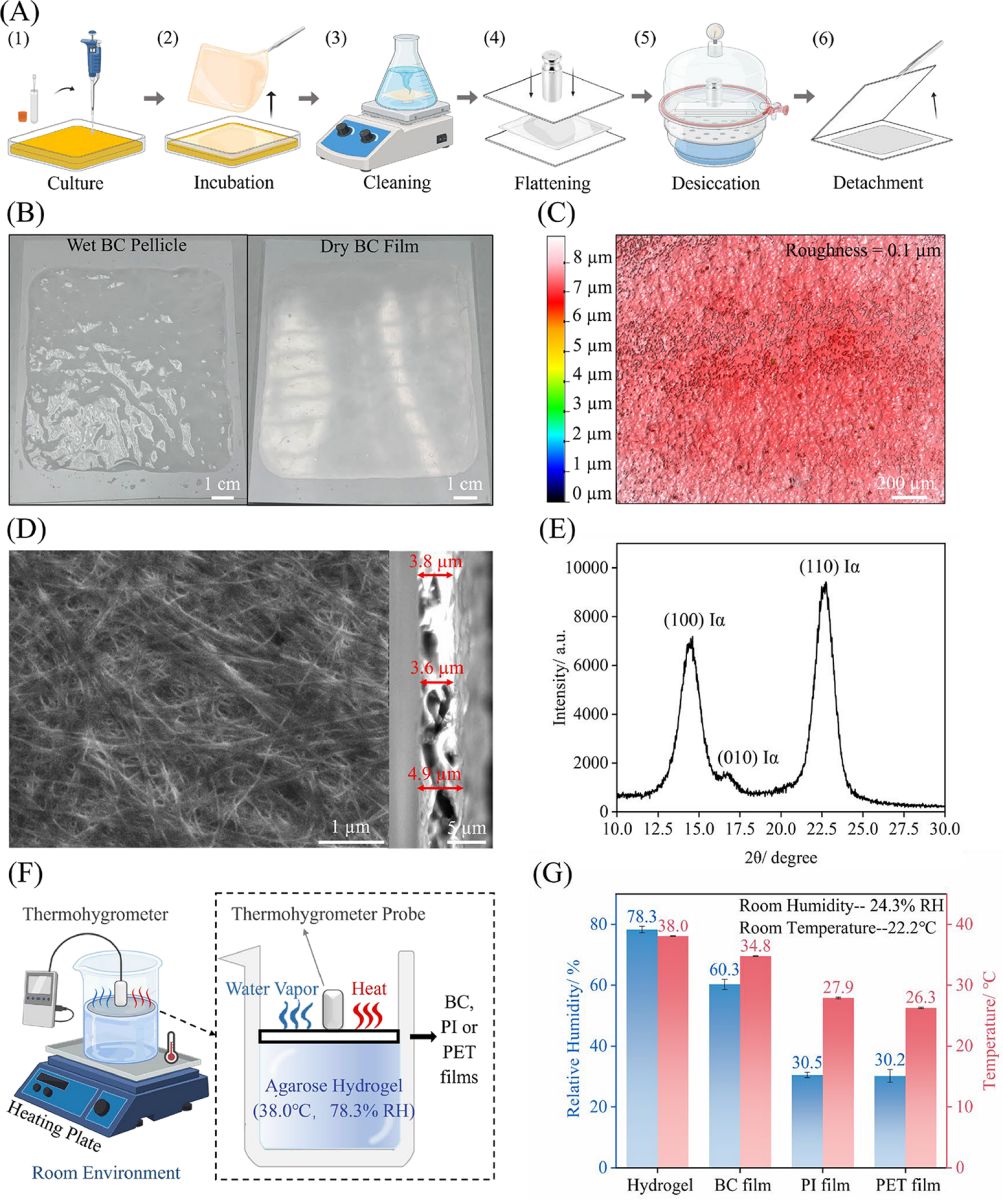
Fabrication and characterization of BC film. (A) The fabrication process of BC film involves the following steps: (1) culture of bacterial strains, (2) incubation of bacteria, (3) cleaning of BC pellicles, (4) flattening of BC pellicles, (5) vacuum drying of BC pellicles, and (6) exposure of dry BC film. (B) Photographs of wet BC pellicles and dry BC film, (C) optical profilometer image of BC film surface, (D) SEM images of the surface and cross-section of BC film, (E) XRD pattern of BC film, (F) schematic diagram of the experimental setup for the characterization of the water vapor permeability and thermal conductivity, (G) water vapor permeability and thermal conductivity measurement results of BC film compared to the agarose hydrogel, PI and PET films (sample number = 10, the error bar represents the standard deviation and the data statistics details are shown in Table S1).

### 2.3 Fabrication of the Sandwiched BC Sensor

The sandwiched BC-based sweat sensor was fabricated using a screen-printing technique. First, a dielectric layer was screen-printed onto the BC film supported on a PET substrate and subsequently cured under UV light for 60 s to act as a waterproof barrier. Next, Ag/AgCl ink was printed and dried at 80 °C for 30 min to form the pseudoreference electrode (RE) and electrical connection. A carbon ink containing PB was then screen-printed and cured at 80 °C for 15 min to define the working electrode (WE) and counter electrodes (CE). A circular WE was designed with a diameter of 1.5 mm. Finally, a second dielectric layer was printed to precisely define the electrode area and isolate the electrical tracks, followed by UV curing for 60 s.

Additionally, the screen-printing process was precisely performed at the center of the BC film. Although the defined printing frame measured 9 × 9 cm^2^, the actual electrode area was only 6 × 8 cm^2^, centered within the frame. Only 12 electrodes were printed on each BC film, ensuring placement within a more homogeneous central region and minimizing the influence of thickness variations near the edges.

### 2.4 Immobilization of the Enzymatic Layer

For the fabrication of the alcohol biosensor, an enzymatic layer was immobilized on the surface of the screen-printed WE. A mixture of alcohol oxidase (AOx) solution, bovine serum albumin (BSA) stabilizer (10 mg/mL in H_2_O), and glutaraldehyde (2.5% in H_2_O) was prepared in a volume ratio of 2:1:1. A 5 μL droplet of this solution was then deposited onto the WE surface and air-dried for 4 h. Subsequently, a 2 μL droplet of 5 wt % Nafion 117 solution was applied to coat the electrode. The modified sensor was then dried overnight at 4 °C in a refrigerator. The sensors can be stored in the dark at −20 °C to maintain their enzymatic activity over an extended period, potentially up to two years.^48^

### 2.5 Characterization Techniques

The morphological characterization of the composite material was performed using a scanning electron microscope (SEM, Auriga, Carl Zeiss) operated at 10–15 kV and a Profilm3D optical profilometer (Scientec Iberica, Spain).

The electrochemical performance of BC sensors was assessed using an Autolab PGSTAT30 potentiostat controlled by NOVA v2.0 software (Metrohm Hispania, Spain). Electrochemical characterization was carried out using cyclic voltammetry (CV) and chronoamperometric techniques. CV measurements were performed in a 0.1 M KNO_3_ solution over a potential range of −0.4 to 0.6 V, with a scan rate of 0.05 V/s and a potential step of 0.1 mV, with the PB being the redox species. The chronoamperometric response was recorded after 1 min of immersion in the test solution by stepping the potential to −0.2 V for 60 s. All potentials were measured against the screen-printed Ag/AgCl pseudoreference electrode. The current recorded at *t* = 60 s was used as the analytical signal, if not stated otherwise. The electrochemical performance of the alcohol BC sensor was further evaluated in phosphate-buffered saline (PBS, pH 7.4) and artificial human sweat (pH 7.4).^49^

## 3 Results and Discussion

### 3.1 Preparation and Characterization of the BC Films

Figure 1A illustrates the fabrication process of the dry BC film and the details of the various preparation steps described in the experimental section (Section 2.2). Figure 1B presents photographs of the bacterial cellulose film before and after drying, demonstrating the transformation from a hydrated, gel-like structure to a smooth, flat dry film. The surface roughness of the dry BC film on PET was assessed using an optical profilometer (Figure 1C). The measurement, taken over a 1.9 × 1.7 mm^2^ area, revealed an average roughness of 0.1 μm. In contrast, a standard drying process without PET resulted in a roughness of 2.5 μm (Figure S1), highlighting the significant improvement in surface smoothness achieved with the drying method. A SEM image of the BC film’s surface (Figure 1D) reveals densely packed nanofibers forming a homogeneous structure, consistent with our previous publications.^40,43^ The three-dimensional overlapping of these nanofibers creates dense, interconnected pores, contributing to the film’s efficient moisture absorption and evaporation. The cross-sectional SEM image (Figure 1D) indicates that the BC film has a thickness that varies between ∼3.5 and 5 μm, which ensures rapid heat conduction. Figure 1E presents the XRD pattern of the BC film, showing three characteristic diffraction peaks at 14.5°, 16.6°, and 22.7° (2θ), which correspond to the (100), (010), and (110) planes of cellulose Iα, respectively.

Figure 1F illustrates the schematic setup for testing the water vapor and heat transfer properties of the BC film. Specifically, a 1 wt % agarose hydrogel in a beaker was heated to 38 °C to simulate the conditions of perspiring human skin in an indoor environment.^50^ A thermohygrometer probe was suspended above the hydrogel and maintained in contact with its surface for 10 min to record the temperature and relative humidity (RH) of the hydrogel. Subsequently, a BC film, cut to match the cross-sectional area of the beaker, was placed atop the hydrogel, and the probe in contact with the BC film recorded the corresponding temperature and humidity values. For comparison, the BC film was replaced with a PI film (0.05 mm thick) and a PET film (0.5 mm thick) of the same size, and the resulting temperature and humidity were similarly measured. As shown in Figure 1G, the agarose hydrogel itself exhibits a RH of 78.3% and a temperature of 38 °C. When covered with the BC film, the recorded values are 60.3% RH and 35 °C, significantly higher than the ambient room conditions but closer to the agarose values, indicating effective transmission of water vapor and heat through the BC film to the external environment. In contrast, the use of PI and PET films results in a sharp decrease in humidity due to their intrinsic impermeability, which substantially limits water vapor transmission. Moreover, the lower temperature observed with PI and PET films, compared to the BC film, underscores the superior thermal conductance of the BC film, attributed to its ultrathin nature. Detailed data statistics and analysis can be found in Table S1. Consequently, the BC film exhibits superior water vapor and thermal transfer capabilities, rendering it a promising material for wearable sensor substrates, which place high demands on thermal regulation and moisture management.

### 3.2 Development of a Sandwiched-Structure BC Sensor with Enhanced Electrochemical Performance

In contrast to conventional top-covered sensor designs, an innovative sandwiched-structure sensor was fabricated on BC film (Figure 2A). Both sensor configurations share common components, including BC film on a PET substrate, a sensing layer, and a top cover dielectric layer, which is printed to define the electrode area exposed to the environment. The key feature of the sandwiched BC sensor lies in the incorporation of a bottom dielectric layer that serves as a waterproof barrier between the electrical tracks of the sensor architecture and the BC film, as illustrated in Figure 2B. The bottom layer has the same design of the top cover, hence the electrode area is printed directly on the BC film while the conductive track area is sandwiched between two dielectric layers.

**Figure 2:**
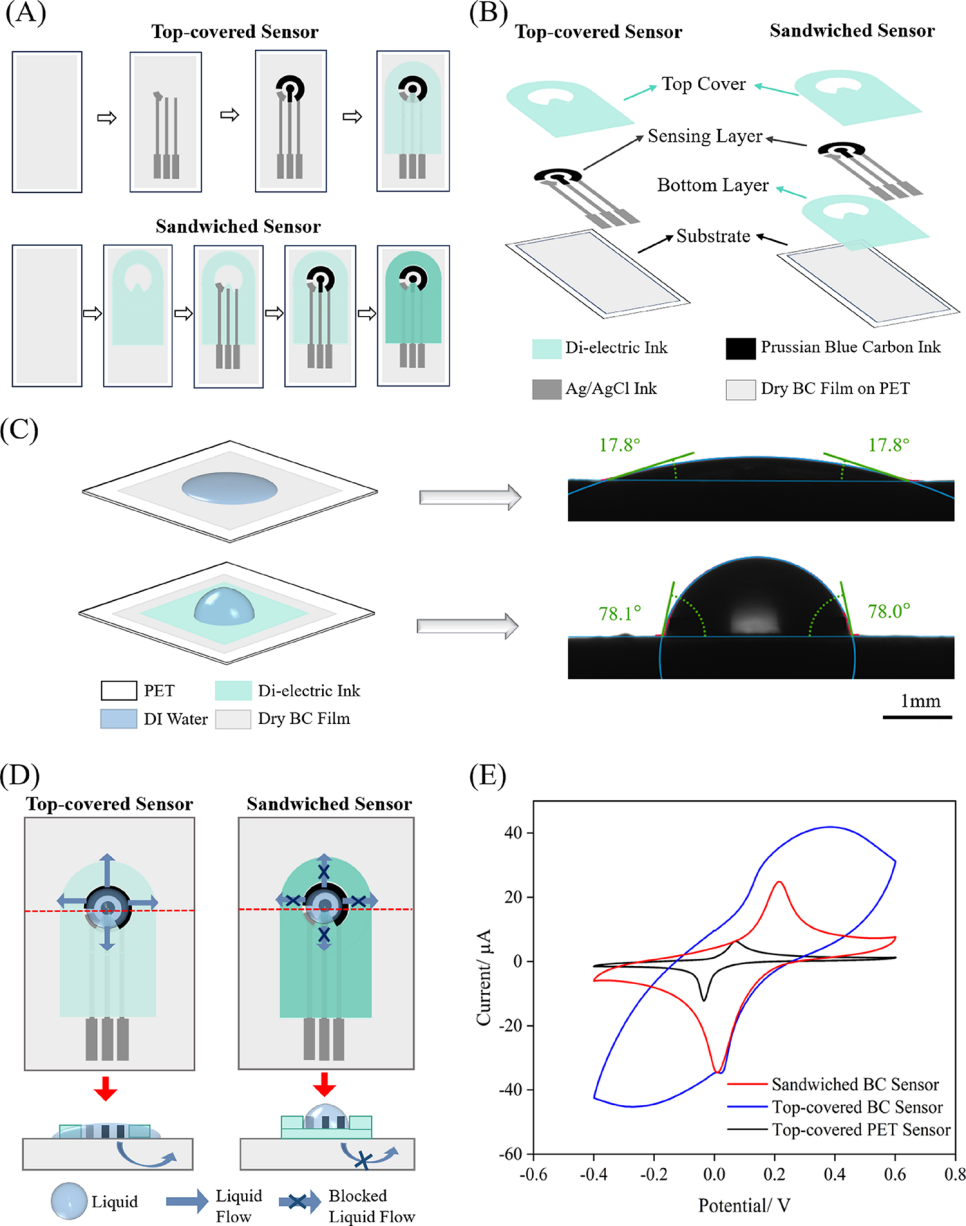
Fabrication and characterization of the BC sensor. (A) Schematic diagram of fabrication steps for the top-covered and sandwiched BC sensor, (B) structure diagram of the top-covered and sandwiched BC sensor, (C) schematic representation and results of the contact angle measurements, (D) schematic representation of the effect of the sandwiched structure compared to the top-covered structure, and (E) CV curves of top-covered PET sensor, top-covered and sandwiched BC sensor. For electrochemical experiment details refer to Experimental Section (Section 2.5).

Figure 2C presents the contact angle measurements of DI water on a pure BC film supported by a PET substrate and on the same construct but coated with a dielectric layer, highlighting differences in surface hydrophobicity of BC compared to the dielectric layer used to insulate the sensor electrical tracks. Additional contact angle data for multiple samples are shown in Figure S2. The pure BC film exhibits extreme hydrophilicity due to the abundance of hydroxyl groups on its surface.^51^ In the top-covered BC sensor, this hydrophilic property causes solutions to permeate the BC film and interact with the electrical tracks beneath the top cover (Figure 2D). Consequently, the effective electrode area extends beyond the defined boundaries of the top cover, leading to an increase in both redox and capacitive currents, as shown in the cyclic voltammetry (CV) curves (blue curve in Figure 2E). The PB redox peaks are masked by high capacitance currents and are difficult to identify. Furthermore, the CV curves of the electrodes demonstrate inconsistencies due to uncertainties in the solution diffusion area (Figure S3). For comparison, the same sensor architecture fabricated on waterproof substrates such as PET exhibits well-defined electrode areas, with reduced redox and capacitive currents due to the absence of solution diffusion (black curve in Figure 2E).

The sandwiched BC sensor addresses this issue by employing a hydrophobic bottom dielectric layer that prevents the solution diffusion, avoiding its contact with the electrodes and electrical tracks outside the defined area (Figure 2D). The contact angle measurements in Figure 2C confirm the hydrophobic nature of the dielectric layer, in contrast to the hydrophilic BC film. In this design, all conductive materials, except for WE, RE, CE areas and the connector pads, are thus isolated by the two layers of dielectric ink to ensure impermeability and avoid electrical contact with external solutions. As a result, the CV curves for the sandwiched BC sensor (red curve in Figure 2E) exhibit reduced capacitive currents and well-defined redox peaks, indicating more consistent electrochemical performance (Figure S4).

Overall, the sandwiched BC sensor demonstrates lower capacitive currents and well-defined redox peaks (red curve in Figure 2E), with anodic and cathodic peak current values of 12.5 and −32.0 μA, which are significantly higher than the 5.5 and −12.2 μA values measured with the top-covered PET sensor, respectively. The peak-to-peak potential of the sandwiched BC sensor is around 0.2 V, also higher than the 0.1 V of the top-covered PET sensor. This increase in both current response and peak-to-peak potential is primarily attributed to the larger effective electrochemical active area provided by the porous and rough morphology of the BC material. Notably, the top-covered BC sensor displays a significantly high capacitive current that masks the faradaic current associated with the Prussian blue redox process. This phenomenon arises from the unconstrained electrode area, a consequence of solution diffusion within the bacterial cellulose to not only the electrodes but most likely to the electrical tracks of the device. The introduction of a sandwich structure effectively mitigates this issue by restricting the solution diffusion pathway, thereby significantly improving the electrochemical performance of the sandwiched BC sensor. From here on and unless stated otherwise, we refer to the sensor fabricated by this approach as sandwiched BC sensor, or simply BC sensor.

### 3.3 Detection Performance of the Alcohol BC Sensor on PET Substrate

Figure 3A illustrates the structure of the alcohol sensor fabricated on BC film. The WE is modified with a mixture of alcohol oxidase (AOx), bovine serum albumin (BSA), and glutaraldehyde, followed by a Nafion coating. BSA and AOx react with glutaraldehyde to produce a network layer firmly attached to the working electrode surface. The Nafion layer serves as a selective barrier, preventing interference from anionic substances such as ascorbic acid or uric acid present in sweat while providing mechanical protection to prevent detachment or dissolution of the modification layer.^52^ The ethanol content in the Nafion layer is more than 1000 times lower than the detection limit of the alcohol BC sensors, and therefore does not interfere with subsequent alcohol measurements (Table S3). Upon exposure to an alcohol-containing solution, the Nafion layer allows the alcohol to diffuse through, where AOx catalyzes the oxidation of ethanol to acetaldehyde and hydrogen peroxide (H_2_O_2_), as represented by the reaction in eq 1).

$${\mathrm{CH}}_{3}{\mathrm{CH}}_{2}\mathrm{OH}\left(\mathrm{ethanol}\right)+O_{2}\overset{\mathrm{AOx}}{\to }{\mathrm{CH}}_{3}\mathrm{CHO}\left(\mathrm{acetaldehyde}\right)+H_{2}O_{2}$$ 1

**Figure 3:**
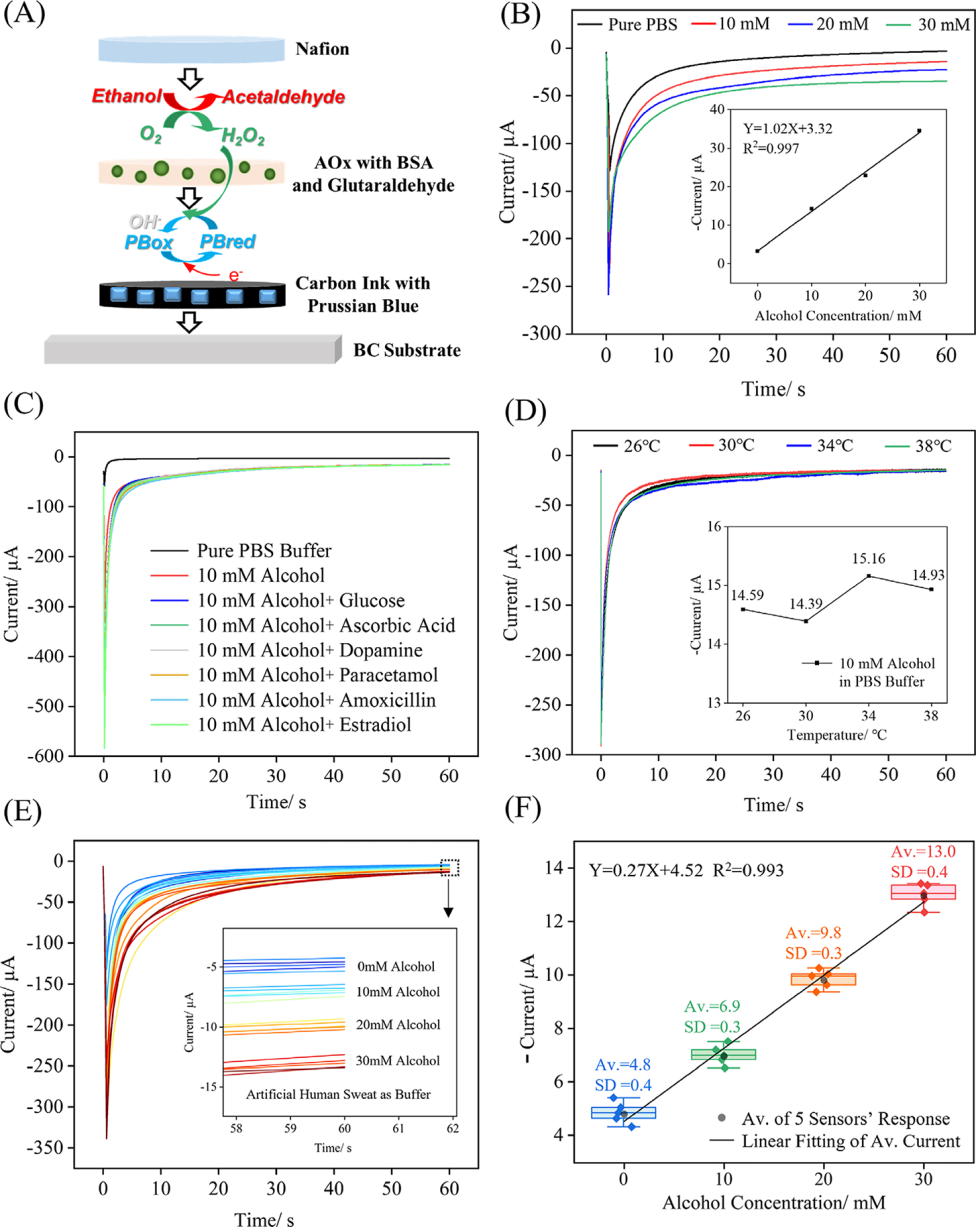
Alcohol detection using the BC sensor. (A) Schematic diagram of the detection principle of the alcohol BC sensor, (B) chronoamperometric responses of alcohol BC sensors in PBS buffer containing alcohol, (C) chronoamperometric responses of alcohol BC sensors in pure PBS buffer, in buffer with alcohol, and in the presence of common physiological interferents, (D) chronoamperometric responses of alcohol BC sensors at different solution temperatures, (E) chronoamperometric responses of four alcohol BC sensor units to different alcohol concentration in artificial human sweat, (F) linear fitting curves of average current to alcohol concentration in artificial human sweat. Av. and SD represent average value and standard deviation of every sensor unit, respectively (sample number = 5 and the data statistic details are shown in Table S3). For electrochemical experiment details refer to Experimental Section (Section 2.5).

PB then catalyzes the reduction of H_2_O_2_ to hydroxide radical, thus lowering the detection potential required to produce a measurable current.^53^ The reaction is shown in eq 2.

$${\mathrm{Fe}}^{3+}\left[{\mathrm{Fe}}^{2+}{\left(\mathrm{CN}\right)}_{6}\right]+H_{2}O_{2}+3e^{-}\to {\mathrm{Fe}}^{2+}\left[{\mathrm{Fe}}^{2+}{\left(\mathrm{CN}\right)}_{6}\right]+2{\mathrm{OH}}^{-}$$ 2

Chronoamperometric measurements were performed and the current magnitude was directly proportional to the H_2_O_2_ concentration, which correlated linearly with the ethanol concentration. Based on the BC sensor’s cyclic voltammetry (red curves in Figure 2E), the PB reaction kinetics indicate that −0.2 V (vs Ag/AgCl pseudoreference) is optimal for chronoamperometry. This potential effectively drives the PB-mediated reduction of H_2_O_2_ while suppressing possible redox signals of common sweat interferents (e.g., ascorbic, uric acids or dissolved O_2_), thus maximizing sensor selectivity and stability.

The chronoamperometric responses of the alcohol BC sensor in alcohol-containing PBS buffer are shown in Figure 3B. The corresponding linear fit presented in the inset reveal excellent linearity between the sensor response and alcohol concentration. To evaluate selectivity, the effects of typical sweat components (glucose, ascorbic acid, dopamine) and common drug metabolites (paracetamol, amoxicillin, estradiol) were examined. As shown in Figure 3C, the addition of 10 mM alcohol induced a pronounced increase in current response, with an approximate value of 11.5 μA, indicating the sensor’s clear and sensitive response to the presence of alcohol. In contrast, the coexistence of these potential interferents produced negligible changes in the chronoamperometric signal compared to that of alcohol alone (detailed data in Table S2), indicating outstanding selectivity of the presented sensor.

The alcohol BC sensor performance under physiologically relevant conditions was also investigated by examining the effects of temperature and pH. Infrared thermography of the sweating human skin surface showed temperatures ranging from approximately 28 to 38 °C (Figure S5), largely consistent with reported human skin temperature ranges of 30–37 °C.^54^ Based on this observation, the chronoamperometric responses of alcohol BC sensors were evaluated using 10 mM alcohol in PBS buffer at four representative temperatures: 26, 30, 34, and 38 °C. As shown in Figure 3D, variations within this temperature window had negligible impact on sensor performance, indicating that the alcohol BC sensor maintains stable functionality under physiologically relevant skin temperatures. The influence of pH, simulating authentic sweat conditions (pH 4.0–7.4),^46^ was assessed in PBS buffers across this range. Well-defined linear fitting curves were obtained for all pH conditions (Figure S6). Sensor sensitivity remained nearly constant between pH 6.0 and 7.4 but decreased markedly below pH 6.0, reflecting the intrinsic pH-dependence of the enzyme catalyst. Therefore, integrating a pH sensor in future on-body applications is important to ensure accurate detection.

The alcohol BC sensor’s reproducibility and robustness were further evaluated in artificial human sweat. In an artificial sweat buffered solution, sensors could detect alcohol concentrations as low as 5 mM (Figure S7). To comprehensively assess the reproducibility of sensors, 20 identical alcohol BC sensors were tested in artificial human sweat buffer solutions containing alcohol concentrations of 0, 10, 20, and 30 mM. Five sensors were used as a unit to repeatedly record the chronoamperometric current produced at one alcohol concentration, and all recorded currents are shown in Figure 3E. Figure 3F shows the consistent responses of five sensors for each of the alcohol concentration tested, with the standard deviation of group results being below 0.4 μA, and relative standard deviations of 7.7, 4.7, 3.3, and 3.0%, respectively. Detailed data statistics and analysis can be found in Table S3 in all cases, and the relative standard deviations varying from 3 to 7%. The average currents of four units exhibit a clear linear relationship with the alcohol concentration. The slope and intercept are similar to those shown in Figure S6, which demonstrates the robustness and reliability of the proposed alcohol BC sensors, in contrast to the inconsistent results of the AOx-modified top-covered BC sensors (Figure S8).

Compared to the slope of 1.02 μA/mM in the PBS buffer, the slope in the artificial sweat buffer significantly decreased to 0.27 μA/mM. This reduction in slope indicates that there is a matrix effect of the artificial human sweat on the sensor response. Artificial human sweat is a complex medium containing different substances such as glucose, lactic acid, ascorbic acid, sodium, potassium, calcium, magnesium and urea,^49^ some of which might adsorb nonspecifically onto the sensor surface, thereby negatively affecting its sensitivity to alcohol. However, the outer Nafion layer serving as a nearly nonporous, negatively charged membrane, effectively minimizes interference by excluding undesired species through electrostatic repulsion and molecular size exclusion, and in combination with the high specificity of the enzymatic reaction, ensures excellent selectivity in real sweat samples.^22^

### 3.4 Detection Performance of the Alcohol BC Sensor without PET Substrate

Figure 4A and Supplementary Video S1 illustrate the detachment process of the BC sensor from the PET substrate. Successful detachment was achieved by immersing the sensor flat in DI water for 5–6 h, which allowed water to penetrate the interface between the BC film and the PET substrate, thus weakening the adhesion. To preserve the structural integrity of the sensor, the detachment was initiated by carefully peeling the carbon electrode side (rather than the Ag electrode side) using tweezers. After 24 h of immersion, the BC film was completely and easily removed from the PET substrate without damage, ensuring the sensor’s structural integrity. The BC sensor maintained its original structure even after more than 24 h of immersion in DI water (Figure 4B), confirming the sensor’s stability and durability under these conditions. Additionally, SEM analysis was used to monitor the morphology of the WE before and after detachment to assess potential structural changes resulting from prolonged immersion (Figure S9). To evaluate the impact of the detachment step on the electrochemical performance, Figure 4C depicts the CV curve of the BC sensor after PET removal. The CV test displays clear redox peaks, indicating that the BC sensor retains its electrochemical activity. However, in some cases, the removal of the PET substrate resulted in a loss of electrochemical signal, likely due to cracks produced on the electrical tracks or damage from mechanical stresses generated during the detachment process. To address this issue, two different strategies were employed for keeping the overall sensor integrity and enhancing production yield (Figure S10 and Video S2).

**Figure 4:**
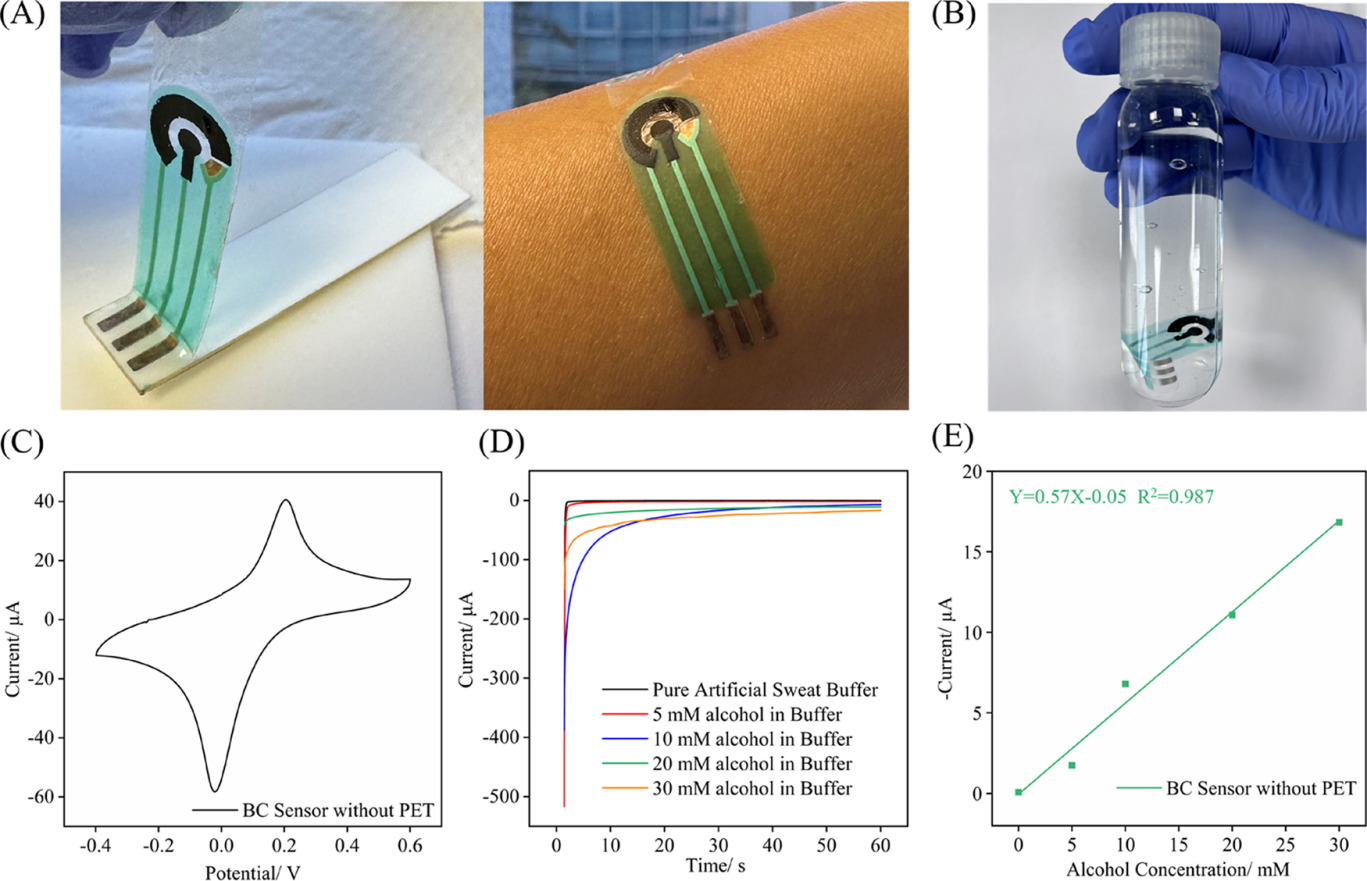
Characterization and performance of BC sensors without PET substrate. (A) Process of detaching the BC sensor from PET substrate, (B) photograph of the BC sensor immersed in DI water, (C) CV curve of the BC sensor after removal from PET substrate, (D) chronoamperometric responses of the alcohol BC sensor without PET substrate, (E) linear fitting curves of the alcohol BC sensor in artificial human sweat spiked with alcohol. For electrochemical experimental details, please refer to Experimental Section (Section 2.5).

The successfully detached BC sensors were subsequently tested for alcohol detection in artificial human sweat under the same conditions as BC sensors on a PET substrate. The electrode modification process remained the same. As shown in Figure 4D, chronoamperometric responses of these sensors were recorded in artificial sweat containing alcohol at concentrations of 0, 5, 10, 20, and 30 mM. The corresponding linear fitting curve in Figure 4E demonstrates a strong linear correlation between the sensor response and alcohol concentration. It can be observed that, in the artificial human sweat, the sensitivity of the BC sensor detached from the PET substrate (slope of the curve in Figure 4E) is higher than that of the sensor that remains attached (slope of the fitting curve in Figure 3F). This increase might be related to the fact that, once detached, both sides of the working electrode are immersed in the buffer solution, effectively doubling the active sensing area and thus resulting in a nearly 2-fold increase in sensitivity. These results confirm that the BC sensor exhibits reliable performance both with and without the PET substrate, highlighting its adaptability and effectiveness in alcohol detection applications.

### 3.5 Electrochemical Performance under Mechanical Deformation

The electrochemical performance of the BC sensor was systematically evaluated under bending conditions to simulate its application on human body regions with curved geometries, such as the head, arm and different wrist positions (Figure 5A). To replicate these bending scenarios, BC sensors were affixed to customized acrylic molds creating different bending angles, then their electrochemical responses were recorded (Figure 5B). The specific bending angles corresponding to the simulated body curvature is detailed in Figure S11.

**Figure 5:**
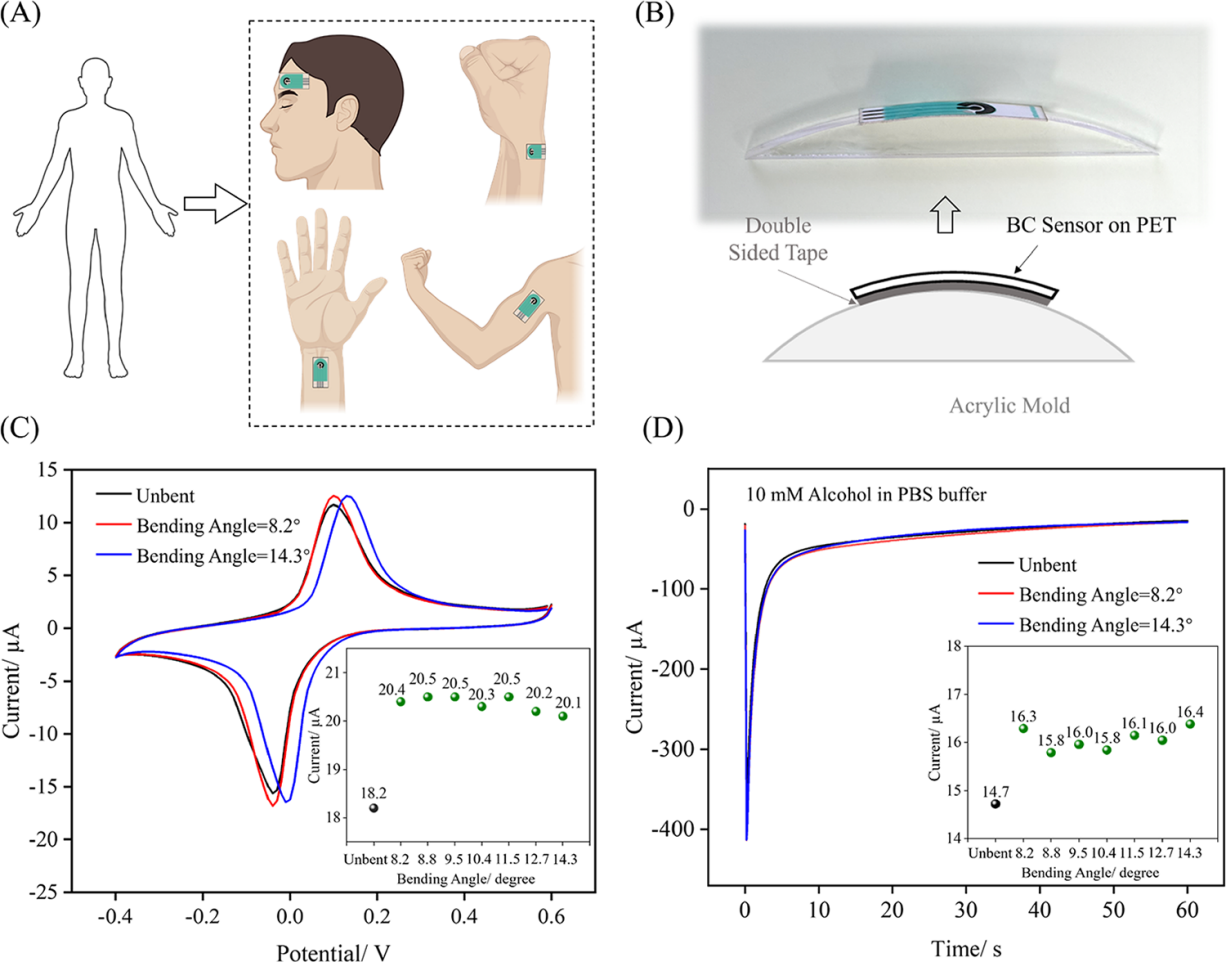
Bending stability of the BC sensor. (A) Schematic illustrating the BC sensor attached to different areas of the curved human body, (B) schematic diagram of the bending test setup for the BC sensors, (C) CV curves of bare BC sensors under flat and bent conditions at angles of 8.2° and 14.3°, with the inset showing the extracted peak current values under detailed bending angles, (D) chronoamperometric curves of alcohol BC sensors under flat and bent conditions at angles of 8.2° and 14.3°, with the inset showing the extracted chronoamperometric values at 60 s under detailed bending angles. For electrochemical experiment details refer to Experimental Section (Section 2.5).

For the bare BC sensors, cyclic voltammetry (CV) measurements were performed to assess the intrinsic electrochemical stability under mechanical deformation. As shown in Figure 5C, whether deformed or not, bare BC sensors exhibit nearly identical redox behavior, indicating that bending did not compromise electrochemical performance. A slight increase in peak current is observed, from 18.2 μA (undeformed) to an average of 20.3 μA (deformed), which can be attributed to bending-induced microcracks in the PB-carbon electrode. These microcracks likely expose additional electroactive sites, thereby enhancing the effective electrochemical active area and, thus, the resulting redox currents. The inset in Figure 5C extracts the peak current values under detailed bending conditions, showing that BC sensor’s peak currents remained relatively stable during the bending test, with a standard deviation of 0.1 μA and a relative standard deviation of 0.5%. These results suggest that although bending induces microcrack formation in the electrode, the extent of cracking does not significantly increase with larger bending angles, thus preserving the electrode’s electrochemical integrity and consistency.

For the alcohol BC sensors, chronoamperometric responses were also recorded under these bending angles in PBS buffer containing 10 mM alcohol to assess their operational stability during alcohol detection. Figure 5D shows similar current profiles under flat and bent states, confirming that bending does not compromise the alcohol sensing performance. The inset of Figure 5D extracts the chronoamperometric values under detailed bending angles. A slight increase was observed, from 14.7 μA in the flat state to an average of 16.1 μA after bending. This trend parallels that shown with bare BC sensors and is likely attributable to microcracks in the PB–carbon layer due to bending, which improves the effective electrochemical active area. The recorded currents of bent sensors exhibited a standard deviation of 0.2 μA and a relative standard deviation of 1.2%. Again, the variation is negligible compared with the 1.6 μA current difference between flat and bent states. These findings confirm that bending does not adversely influence the chronoamperometric response, thereby demonstrating that the alcohol BC sensors maintain stable and reliable functionality under mechanical deformation.

### 3.6 Eco-Friendly and Skin-Compatible Adhesion of the BC Sensor

Figure 6A shows photographs of the BC sensor applied to the skin via its front surface, a common configuration in wearable sweat sensors for real-time monitoring. In this setup, the electrodes directly interface with the skin, enabling the detection of target analytes upon sweat secretion. To ensure tight conformal contact between the electrodes and the skin, an additional adhesive layer is typically introduced. However, sweat gradually accumulates between the sensor and the skin, which weakens the adhesion and presents challenges for wearability and long-term stability. Moreover, prolonged use of adhesive layers, even those made of medical-grade tapes, can lead to skin irritation, including redness, itching, and discomfort (Figure 6D).

**Figure 6:**
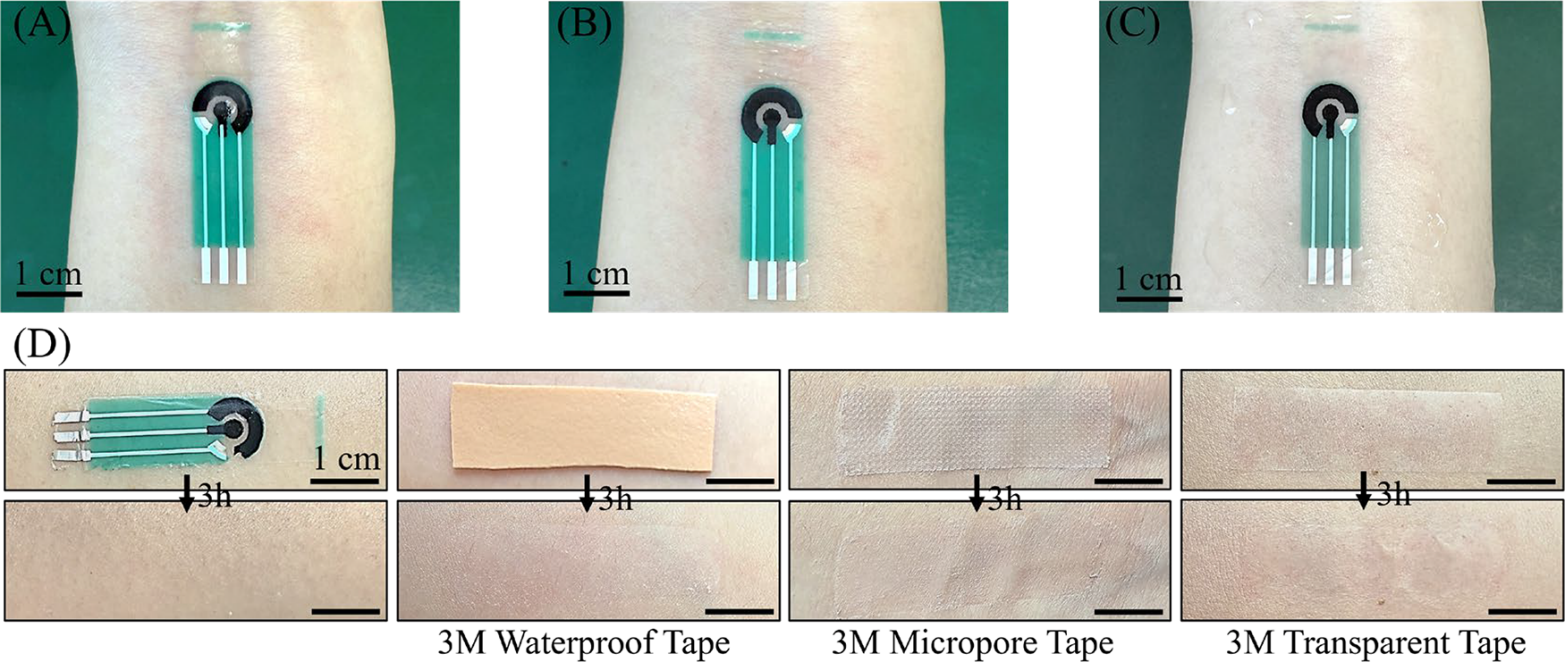
BC sensor contact with human skin. (A) Photograph showing the frontside contact of the BC sensor with human skin, (B) photograph showing the backside contact of the BC sensor with human skin, (C) photographs of the BC sensor backside contacts with wet human skin, (D) no skin reaction was observed 3 h after the BC sensor’s backside contact with human skin vs skin reaction observed 3 h after other adhesive material’s contact with human skin.

In contrast, the BC sensor leverages the exceptional hydrophilicity of its BC substrate, enabling strong adhesion to the skin on both the frontside and backside surface without requiring adhesive materials (Figure 6A,B and Videos S3, S4). The self-adhesive property clearly simplifies the sensor configuration and fabrication steps. The backside of the sensor consists of pure BC film, which maintains robust adhesion even as sweat accumulates (Figure 6C). Even during daily activities or prolonged exercise, the BC sensor remains securely attached and presents a great tensile strength (Figure S12). The porous nature of the BC material facilitates efficient vertical liquid transport (Video S5), ensuring optimal contact with the frontside electrode for real-time, accurate sweat composition analysis. This innovative backside-contact approach also broadens the possibilities for developing advanced sweat sensor architectures.

Additionally, BC outstanding breathability and thermal conductance contribute to enhanced skin comfort. Even after 3 h of continuous wear, no signs of irritation, such as redness or discomfort, are observed (Figure 6D). Its biocompatibility allows users to wear it for extended durations without physiological discomfort, even if they forget to take it off. This is especially convenient when users are busy, exercising vigorously, or have limited mobility. This feature also makes BC sensors ideal for manufacturing wearable sensors used for long-term continuous application on the human body. Furthermore, BC degradation ensures that once discarded, it degrades naturally without causing environmental pollution. To evaluate its biodegradability, a BC sensor was immersed in a low-concentration (1 mM) cellulase enzyme solution in water. Over the course of one month, the BC film gradually dissolved (Figure S13), in a rather slow process attributed to its compact nanofiber structure formed during the drying process. By contrast, conventionally dried BC films without PET substrate, which have a less compact structure, dissolved much faster—within approximately 1 week under the same conditions. These findings highlight the BC sensor potential for long-term, user-friendly applications in personalized healthcare while promoting sustainability through its environmentally friendly decomposition.

## 4 Conclusions

This study presents an eco-friendly, conformal and self-adhesive wearable electrochemical sweat sensor using bacterial cellulose (BC) film as the sensor substrate and showing robust and reproducible electrochemical performance. The BC-based sensor, fabricated by screen printing sandwich-structured electrodes onto dry BC film, integrates easy operation, user comfort, and environmental sustainability. Compared to commonly used sensor substrate materials, BC film demonstrates superior conformability, gas and liquid permeability as well as thermal conductivity, significantly enhancing wearing comfort. The inherent self-adhesiveness, biocompatibility, and permeability of BC film eliminate the need for external adhesives, ensuring both convenience and long-term stability during sensor wear, with no observed skin irritation even after 3 h of use. Additionally, the sensor’s bidirectional functionality opens new avenues for innovative sweat-sensing system designs. The biosynthetic and biodegradable nature of the BC substrate further enhances its environmental appeal, as it can naturally degrade after disposal. Crucially, the novel sandwich structure effectively addresses the issue of unstable solution permeation caused by the hydrophilicity of BC films, ensuring low capacitive currents and stable electrochemical performance. This BC sensor maintains a consistent electrochemical response at bending angles ranging from 8.2° to 14.3°. The sweat alcohol sensor based on BC exhibits a highly linear response to alcohol concentration in artificial human sweat, with a minimum detection concentration of 5 mM and excellent reproducibility. The successful demonstration of sweat alcohol detection highlights the sensor’s practical applicability and its potential for accurately monitoring various low-concentration biomarkers in sweat. The primary objective of this work is to establish a solid technical foundation for the design and fabrication of BC-based sensors. Moving forward, we intend to develop an on-body BC-based sweat sensing platform, integrating additional sensor arrays—including temperature, pH, and sweat rate sensors—to enable more accurate and comprehensive biochemical analysis. This work not only advances the broader applications of BC in wearable biosensing but also contributes to the broader objective of sustainable wearable technologies by demonstrating that high-performance biosensors can be engineered through an eco-conscious design strategy.

## Supplementary Material













## Notes

Additional information about roughness characterization of BC film without PET; contact angles of pure BC film samples and BC films coated with dielectric ink samples; electrochemical performance stability of sandwiched BC sensor versus top-covered BC sensor; infrared thermal image of a volunteer’s sweating skin surface; alcohol BC sensors performance in PBS buffer with various pH conditions; alcohol BC sensors performance in artificial human sweat buffer; performance of AOx-modified sandwiched BC sensor versus top-covered BC sensor; morphology characterization of the BC Sensor before and after removal from PET; strategies for the BC sensor removal to enhance its signal; conversion relationship between bending angles and human chest curve; tensile strength of the BC sensor; biodegradability of the BC sensor; data statistics and analysis of temperature and humidity measurement results; data statistics and analysis of chronoamperometric responses of BC alcohol sensors in interferent experiments; data statistics; and analysis of chronoamperometric responses of BC alcohol sensors in artificial human sweat buffer (PDF)
Entire BC sensor removal from PET substrate (MP4)
Half of BC sensor removal from PET substrate (MP4)
BC sensor’s frontside attachment on the arm-electrodes in direct contact with skin (MP4)
BC sensor’s backside attachment on the arm-electrodes in indirect contact with skin (MP4)
Vertical liquid permeability of pure BC film (MP4)
