# Supplementary material for: Eco-Friendly Conformal and Self-Adhesive Electrochemical Sensors for Sweat Monitoring
Source: ACS Appl Mater Interfaces. 2025 Sep 16;17(39):54411–22. doi: 10.1021/acsami.5c07032 (PMC12492337; doi:10.1021/acsami.5c07032)
Supplement: Supplementary file 1 [file am5c07032_si_001.pdf]

Supporting Information

Eco-friendly Conformal and Self-adhesive Electrochemical Sensors for Sweat Monitoring

Xiaohe Wang[1,2], Muling Zeng[2], Mabel Torrens[3], Pengfei Niu[1]*, César Fernández Sánchez[3,4]*, Marti Gich[2]*and Anna Roig[2]*

1. State Key Laboratory of Precision Measuring Technology & Instruments, Tianjin University, Tianjin 300072, China
2. Institut de Ciència de Materials de Barcelona, ICMAB (CSIC), Campus UAB, 08193 Bellaterra, Spain
3. Instituto de Microelectrónica de Barcelona, IMB-CNM (CSIC), Campus UAB, 08193 Bellaterra, Spain
4. Centro de Investigación Biomédica en Red de Bioingeniería, Biomateriales y Nanomedicina (CIBER-BBN), 28029 Madrid, Spain

Corresponding authors

*E-mail: niupengfei2018@tju.edu.cn
*E-mail: cesar.fernandez@csic.es
*E-mail: mgich@icmab.es
*E-mail: roig@icmab.es

Contents

Figures 3

Figure S1. Optical profilometer image of the dry BC film surface with a standard drying process without PET 3

Figure S2. Contact angles of three pure BC film samples and three BC films coated with dielectric ink samples 4

Figure S3. CV curves of the three top-covered BC sensor devices in 0.1M KNO_3 4

Figure S4. CV curves of three sandwiched BC sensor samples in 0.1M KNO_3 5

Figure S5. Infrared thermal image of a volunteer's forearm during exercise-induced sweating 6

Figure S6. Chronoamperometric responses of alcohol BC sensors in PBS buffer with various pH conditions 7

Figure S7. Chronoamperometric responses of alcohol BC sensors in artificial sweat 8

Figure S8. Chronoamperometric responses of AOx modified top-covered BC sensors9

Figure S9. SEM images of the working electrode of BC sensor 10

Figure S10. Two different strategies to address the connectivity to the potentiostat 11

Figure S11. Schematic diagram for calculating the bending angle of a simulated human chest curve 12

Figure S12. Tensile stress-strain curve of BC sensor 13

Figure S13. BC sensor before and after immersion in cellulase enzyme solution 13

Tables 14

Table S1. Data statistics and analysis of temperature and humidity measurement results 14

Table S2. Data statistics and analysis of chronoamperometric responses of BC alcohol sensors in pure PBS buffer, in buffer with alcohol, and in the presence of common physiological interferents. 15

Table S3. Data statistics and analysis of chronoamperometric responses of BC alcohol sensors in artificial human sweat containing alcohol concentrations of 0, 10, 20, 30 mM, respectively. 16

References 17

Figure S1. Optical profilometer image of the dry BC film surface with a standard drying process without PET.

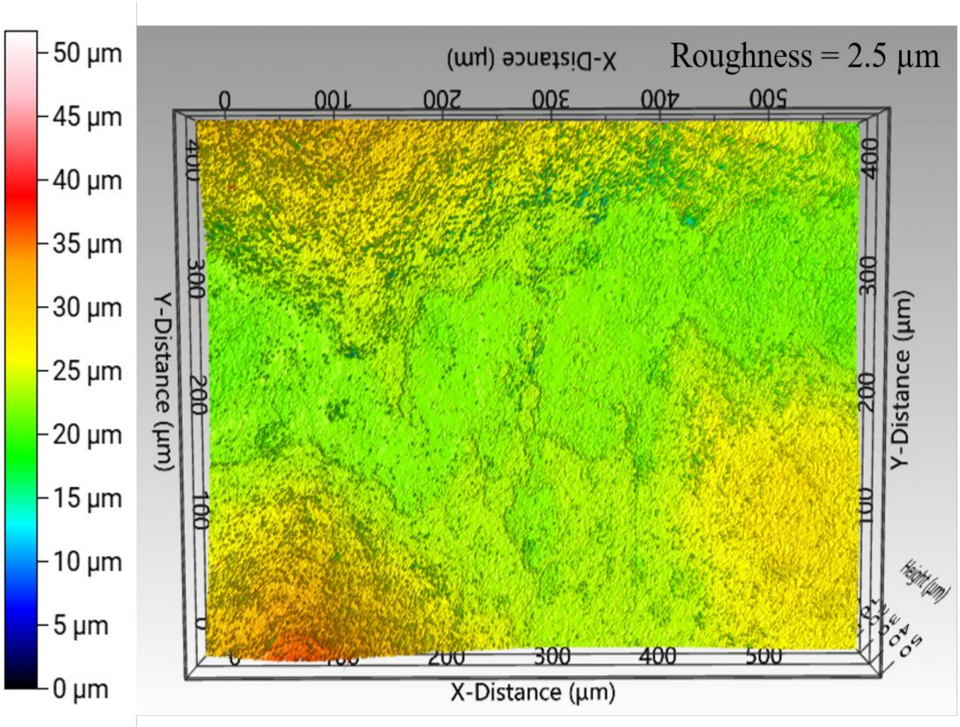

A standard drying process follows these steps: Cleaned wet BC pellicles (10 x 10 cm) are sandwiched between two Teflon sheets, subjected to a 2 kg weight, and dried under vacuum until fully desiccated. Finally, the two Teflon sheets can be removed easily, yielding a dry but rougher BC film. The roughness of the optical profilometer image, taken over a 0.6 mm ×0.4mm area, for this standard drying process is around 2.5 μm, much higher than that of a dry BC film using a PET substrate (0.1 μm).

Figure S2. Contact angles of three pure BC films (a,b,c) and three BC films coated with dielectric ink samples (d,e,f). Contact angles are included in the images.

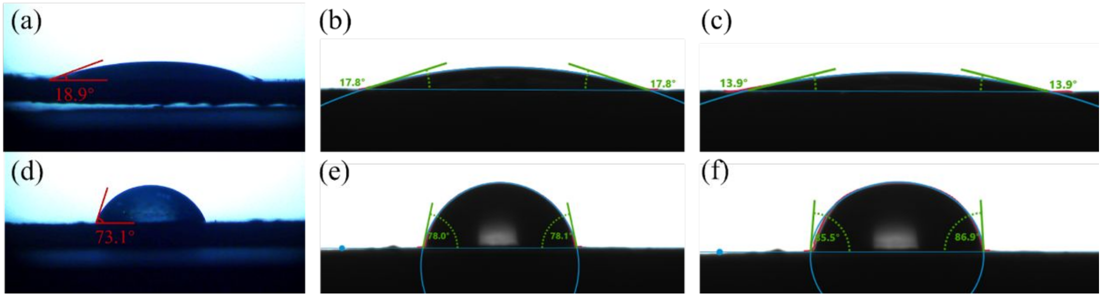

Figure S3. CV curves for three different top-covered BC sensors in 0.1M KNO_3. The CV curves of the three top-covered BC sensors display poor consistency and high capacity current because of the irregularities and differences between replicates in the solution diffusion area beneath the top cover and KNO_3 solution interaction with the undefined conductive inks area.

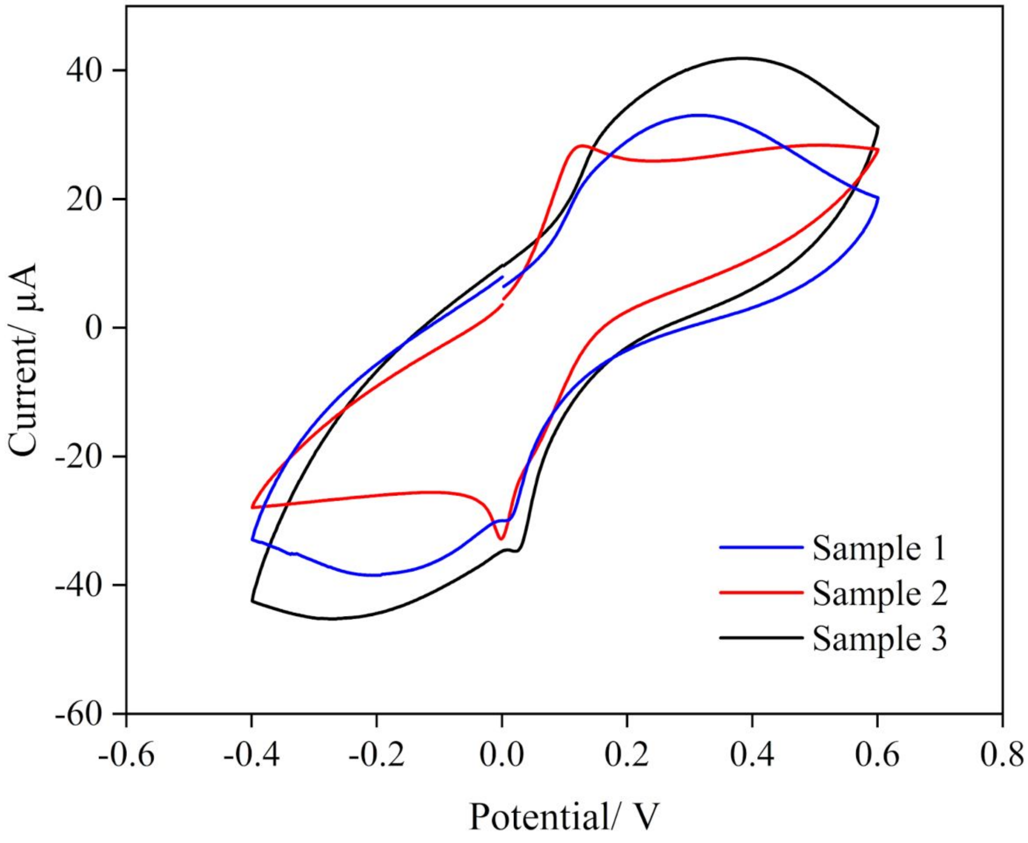

Figure S4. CV curves of three different sandwiched BC sensors in 0.1M KNO_3 displaying excellent consistency and a clear redox current due to the KNO_3 solution interaction with a well-defined conductive area.

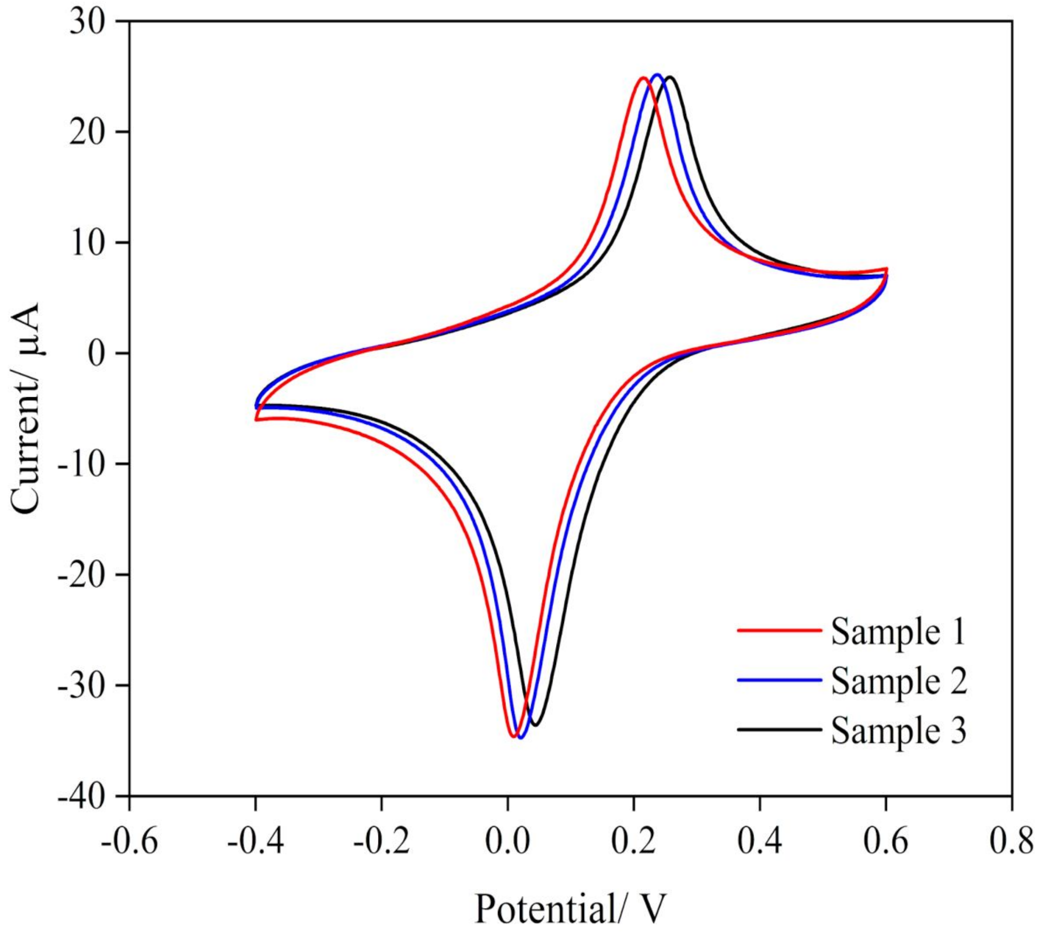

Figure S5. Infrared thermal image of a volunteer's forearm during exercise-induced sweating.

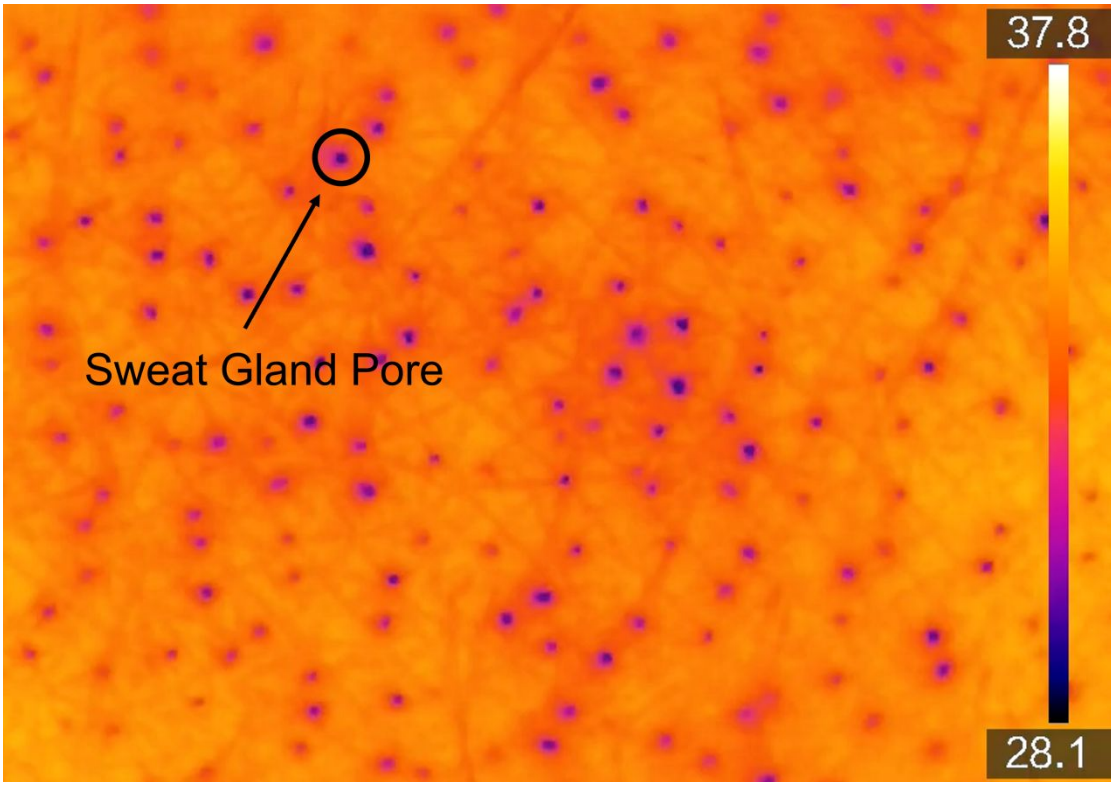

The minimum temperature, approximately 28 °C, was observed at the locations of sweat gland pores. This local cooling effect is attributed to the endothermic nature of sweat evaporation, which removes heat from the skin surface and results in a lower temperature at these sites compared with the surrounding skin.

Figure S6. Chronoamperometric responses of alcohol BC sensors in PBS buffer with different pH values.

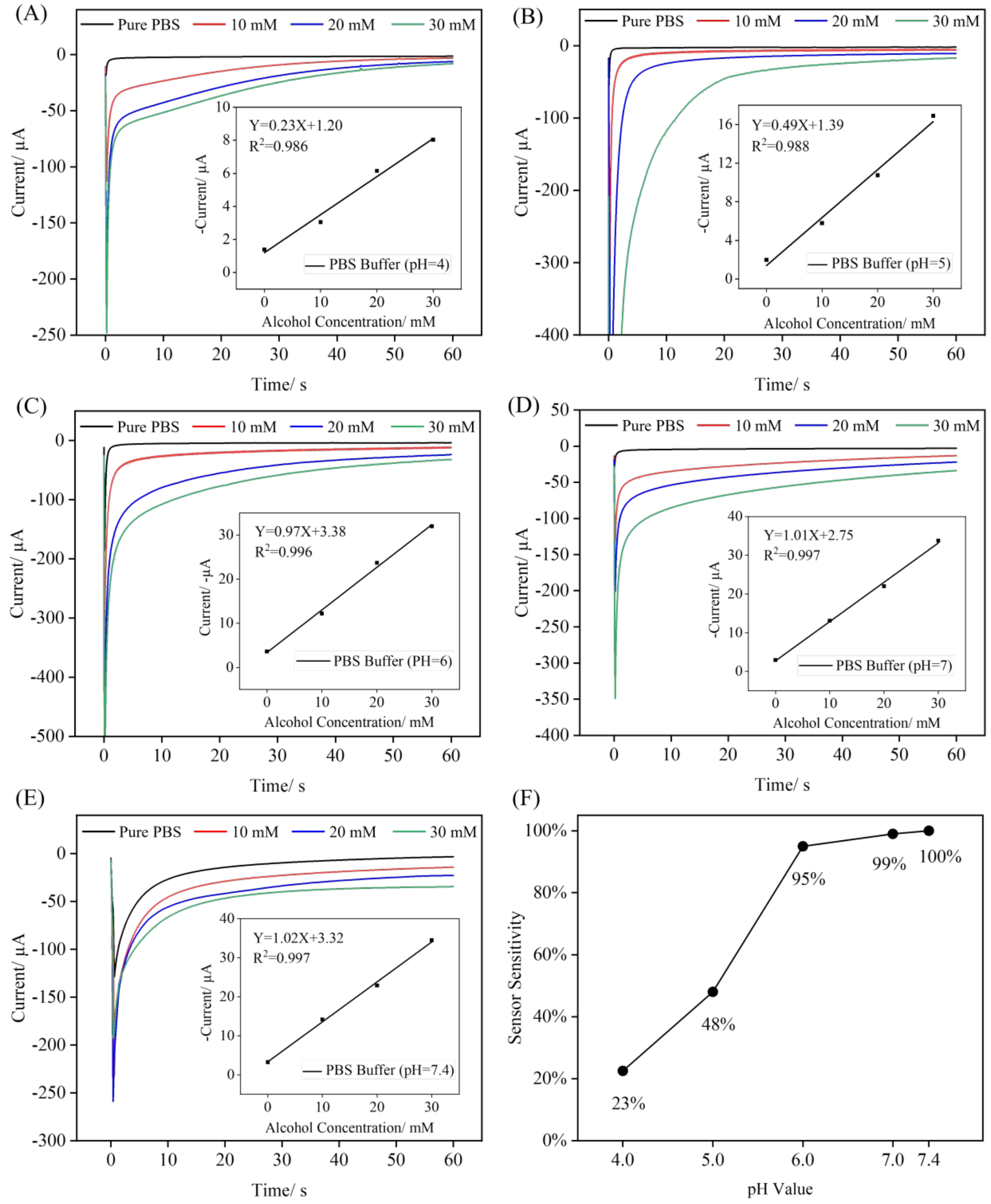

Different concentrations of alcohol were added to PBS buffers with various pH values, and the chronoamperometric responses of alcohol BC sensors exhibited good linearity across all conditions (Figure S6 (A-E)). The slope of the linear fitting curve is commonly used to evaluate the sensor sensitivity. Therefore, the slope at pH 7.4 was taken as the reference value, and slopes at other pH values were normalized accordingly, as shown in Figure S6 (F). It can be seen that when the buffer pH ranged from 6 to 7.4, the sensor sensitivity remained nearly constant. When the pH dropped below 6, the sensitivity began to decrease. Human sweat pH varies under different physiological conditions. Specifically, the pH of primary sweat is about 7.1-7.4, but the pH value can vary after sweat reaches the skin surface. Bicarbonate reabsorption

in the sweat gland duct is inversely related to sweat rate. Thus, at low sweat rates, the primary sweat is exposed to the sweat gland duct for a long time and is further acidified, which results in a pH of about 4-5. However, pH can remain as high as 6.9 at quicker flow rates[1]. Therefore, pH calibration is not required under natural sweating conditions, such as during intense exercise or high environmental temperature. In contrast, pH calibration becomes necessary under light exercise conditions, where small pH changes can influence sensor readings. Therefore, integrating a pH sensor in future on-body applications is important to ensure accurate signal calibration under such scenarios.

Figure S7. (A) Chronoamperometric responses of alcohol BC sensors in artificial human sweat buffer, (B) Linear fitting curve of chronoamperometric values at 60s.

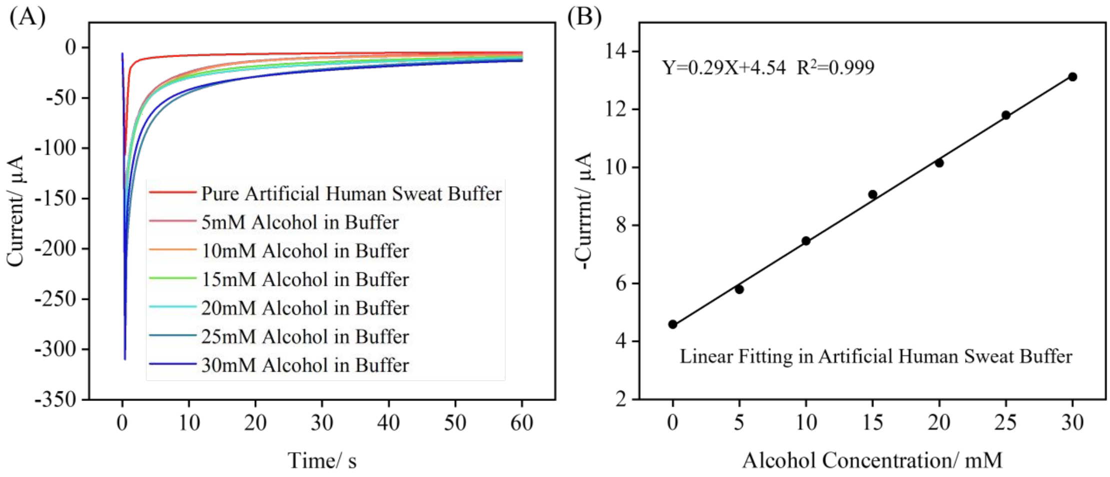

Figure S8. Chronoamperometric responses of AOx modified top-covered BC sensors recorded in PBS buffer containing 0, 10, 20 and 30 mM alcohol. The absolute values of the current results are 24.9, 44.2, 39.1 and 46.6 μA, respectively. No clear correlation between alcohol concentration and current magnitude is observed.

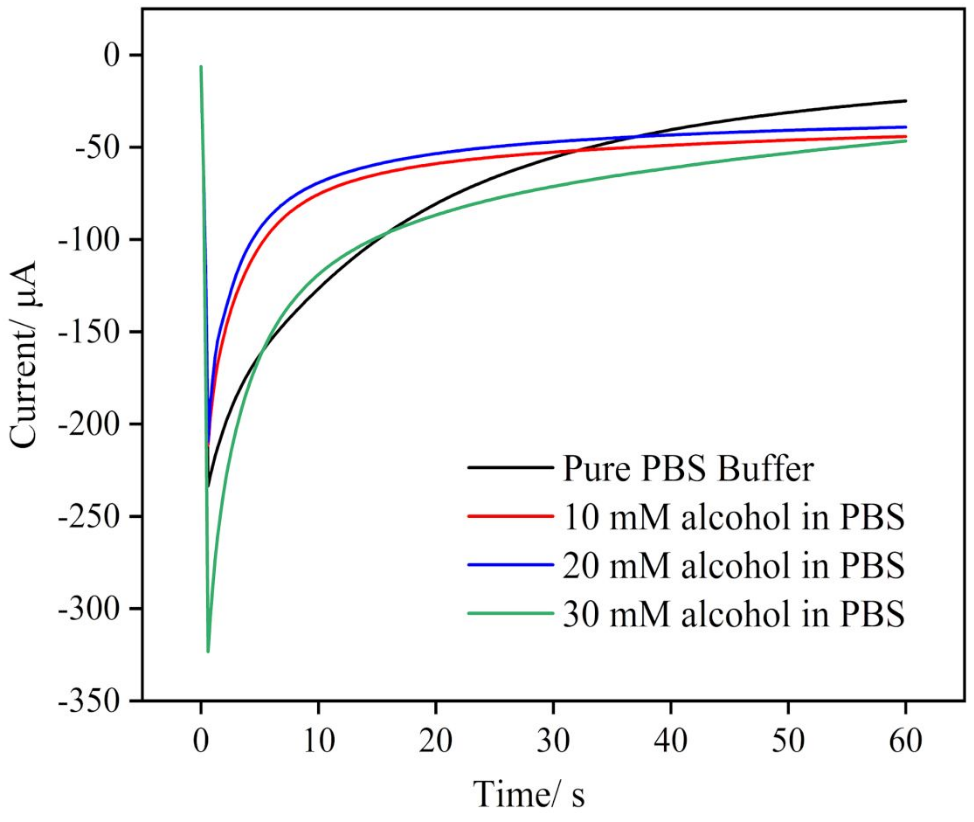

Figure S9. SEM images of the working electrode (WE) of BC sensor. (A) Four positions of the WE were selected for SEM analysis to visualize the WE morphology. SEM Images of BC sensor before (B) and after (C) removal from the PET substrate. Positions 1-4 correspond to the locations marked in Figure 4A, showing the same surface morphology of the BC sensor post-detachment. The morphology of the working electrode did not change significantly after 24 h of immersion and the peeling-off process.

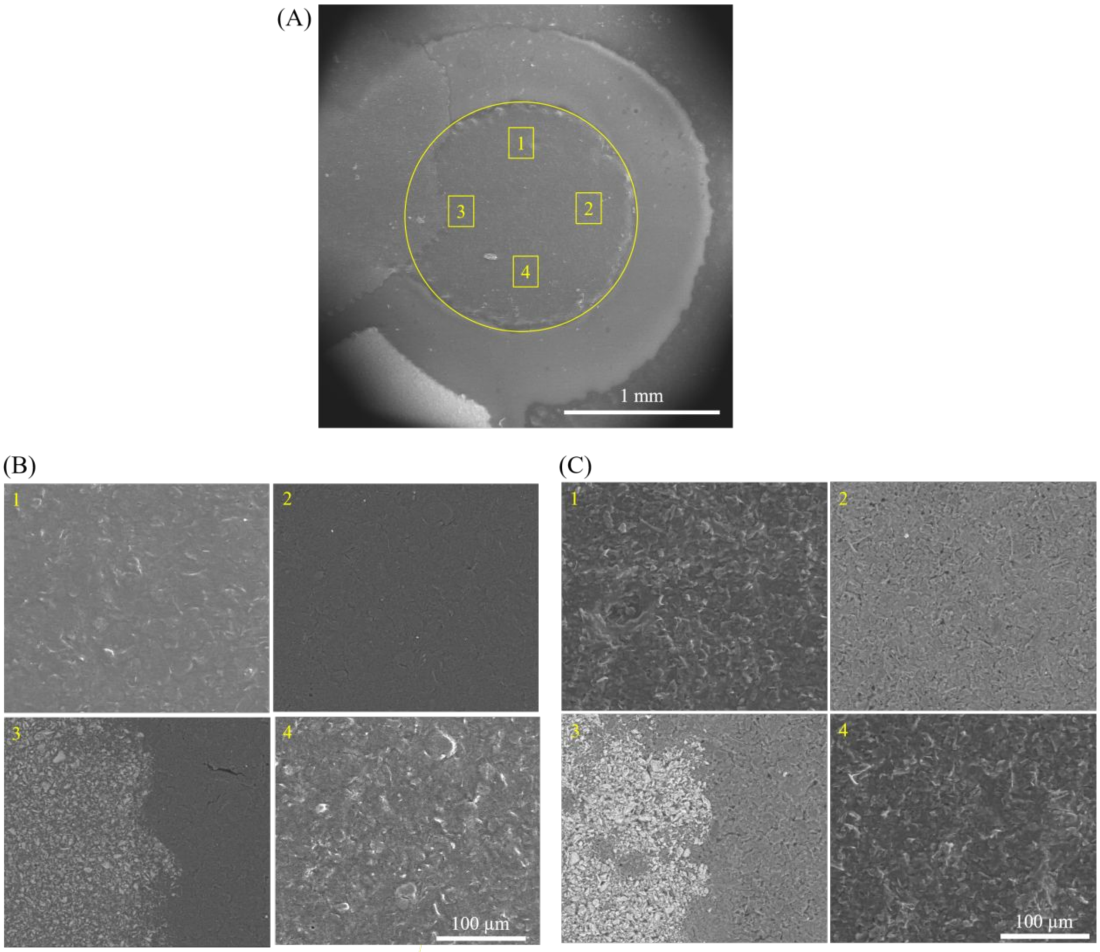

Figure S10. Two different strategies to address the connectivity to the potentiostat. (A) Partial detachment of the BC sensor, removing only the carbon electrode while keeping the Ag electrode on the PET substrate; (B) Application of an additional Ag layer over the existing Ag electrodes to enhance conductivity and signal stability.

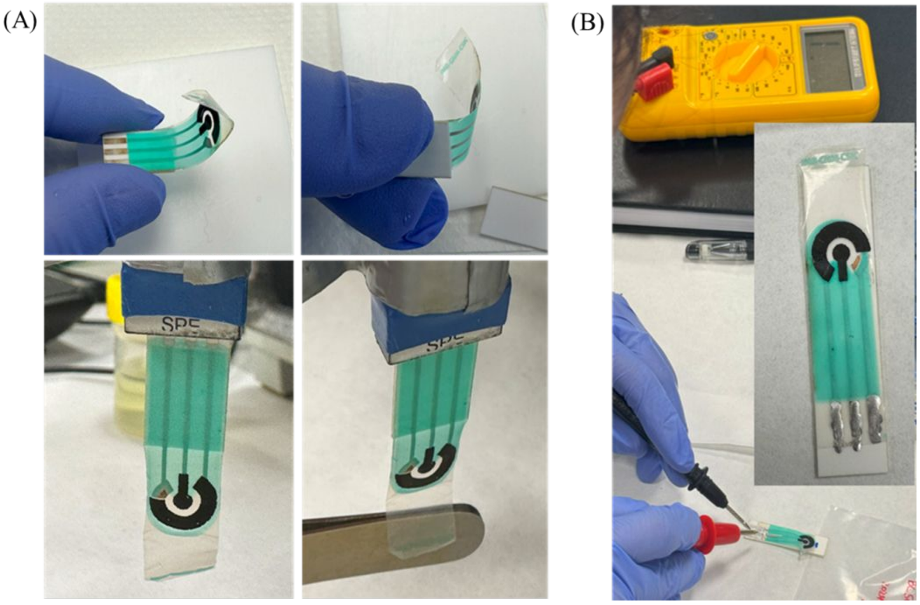

Instead of fully detaching the BC sensor, only the portion containing the carbon electrode was removed from the PET substrate, leaving the half with the Ag electrical tracks intact, while scissors were used to carefully trim away the remaining PET substrate. This method helped preserve the connector pad integrity and reduce the risk of damage. Additionally, to further improve conductivity and signal stability, an extra layer of Ag was applied over the existing Ag electrodes to reinforce the electrical pathways.

Figure S11. Schematic diagram for calculating the bending angle of a simulated human chest curve.

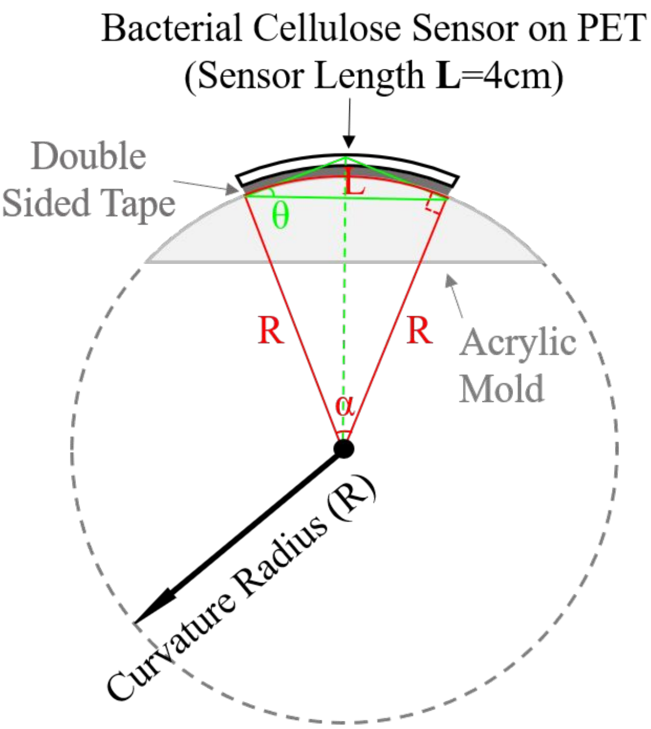

The radius of curvature of the human body chest varies from 80 to 140 mm.[2] To simulate the curve of the human chest, acrylic molds with a radius of curvature (R) ranging from 80 mm to 140 mm are fabricated via CO_2 laser cutter. The BC sensor is attached to the curved acrylic surface with double-sided adhesive tape, replicating its adhesion to the human body. Then the performance of the bent sensor is tested. To enhance clarity, the bending angle (θ) was used instead of the radius of curvature to quantify the degree of bending. The bending angle (θ) is defined as the angle between the tangent line at the sensor's endpoint and the horizontal plane. Additionally, α denotes the central angle corresponding to the sensor's length. The relationship between the bending angle (θ) and the radius of curvature (R) is described by the following conversion formulas:

$$\theta = \frac{\alpha}{2} = \frac{\frac{L}{2\pi R} \times 360^\circ}{2}$$

The radius of curvature of the acrylic model is adopted as 80 mm, 90 mm, 100 mm, 110 mm, 120 mm, 130 mm and 140 mm. The corresponding sensor bending angles are 8.2°, 8.8°, 9.5°, 10.4°, 11.5°, 12.7° and 14.3°, respectively, as shown in Figure 5C and 5D.

Figure S12. Tensile stress-strain curve of BC sensor. The tensile strength of a BC sensor without PET was measured using a tensile tester (Jipin Techniques, Mark-10, China). Tensile test results in Figure S8 reveal that the BC sensor breaks when stretched to 1.6 mm, with a tensile force of 3.612 N at the breaking point. The BC sensor has a tensile strength of 19.02 MPa and a breaking elongation percentage of 40 %.

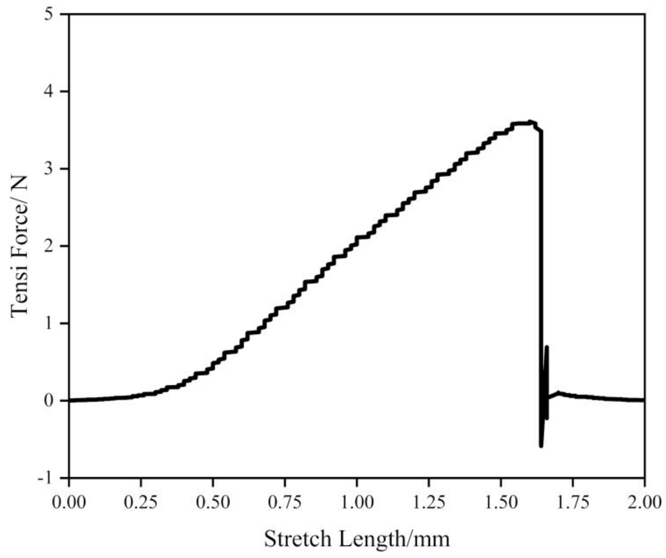

Figure S13. BC sensor before (A) and after (B) one month of immersion in a low-concentration (1 mM) cellulase enzyme solution in water, demonstrating the gradual biodegradation of the BC film over time.

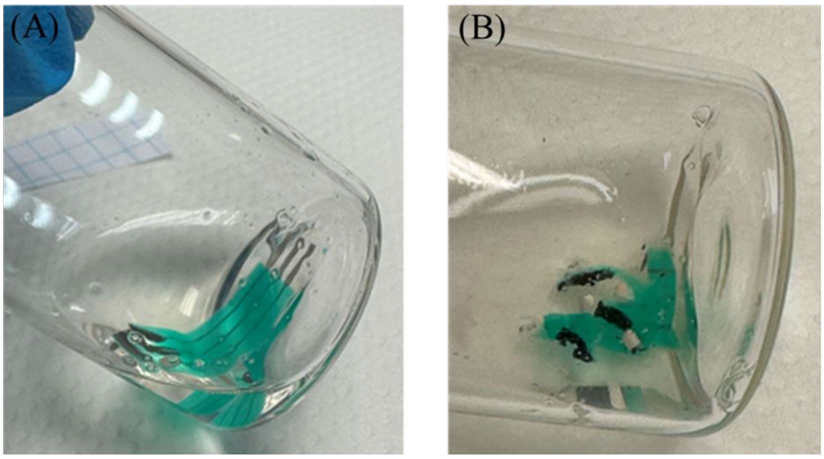

Table S1. Data statistics and analysis of temperature and humidity measurement results. The thermohygrometer records the relative humidity value and temperature value every minute. The recording time lasts ten minutes; therefore, the number of measurements for each set of temperature and humidity is 10 (n=10). Twenty BC alcohol sensors were used to record the chronoamperometric currents in an artificial human sweat buffer. Five samples are used for each alcohol concentration. Sample 1, 2, 3, 4 and 5 were tested in pure artificial human sweat. Sample 6, 7, 8, and 10 were tested in artificial human sweat containing 10mM alcohol. Sample 11, 12, 13, 14 and 15 were tested in artificial human sweat containing 10mM alcohol. Sample 16, 17, 18, 19 and 20 were tested in artificial human sweat containing 10mM alcohol. The sample number for each dataset (n) is 5.

Recording Time	Humidity Hydrogel	Humidity BC film	Humidity PI film	Humidity PET film	Temperature Hydrogel	Temperature BC film	Temperature PI film	Temperature PET film
1 min	78.8 %	60.4 %	30.0 %	30.9 %	37.9 ℃	34.8 ℃	28.1 ℃	26.5 ℃
2 min	77.3 %	60.6 %	32.0 %	31.0 %	37.9 ℃	34.8 ℃	28.0 ℃	26.4 ℃
3 min	76.8 %	60.7 %	29.9 %	29.2 %	38.0 ℃	34.8 ℃	28.0 ℃	26.4 ℃
4 min	77.3 %	60.8 %	28.9 %	26.9 %	38.0 ℃	34.8 ℃	27.9 ℃	26.4 ℃
5 min	78.2 %	61.9 %	30.7 %	28.8 %	38.0 ℃	34.7 ℃	27.9 ℃	26.3 ℃
6 min	78.2 %	62.4 %	30.6 %	33.1 %	38.0 ℃	34.8 ℃	27.8 ℃	26.2 ℃
7 min	79.9 %	58.1 %	30.5 %	33.8 %	38.0 ℃	34.8 ℃	27.8 ℃	26.2 ℃
8 min	79.5 %	57.0 %	31.1 %	30.1 %	38.0 ℃	34.9 ℃	27.7 ℃	26.2 ℃
9 min	77.9 %	59.7 %	30.4 %	28.5 %	38.0 ℃	34.9 ℃	27.7 ℃	26.2 ℃
10 min	79.5 %	61.2 %	31.2 %	30.0 %	38.0 ℃	34.8 ℃	27.7 ℃	26.2 ℃
**Average Value**	**78.3 %**	**60.3%**	**30.5 %**	**30.2 %**	**38.0℃**	**34.8℃**	**27.9 ℃**	**26.3 ℃**
**Standard Deviation**	**1.06 %**	**1.65 %**	**0.84 %**	**2.09 %**	**0.04 ℃**	**0.06 ℃**	**0.14 ℃**	**0.12 ℃**

Table S2. Data statistics and analysis of chronoamperometric responses of BC alcohol sensors in pure PBS buffer (pH=7.4), in buffer with alcohol, and in the presence of common physiological interferents.

Test Solution	Chronoamperometric Value at 60 s (μA)
Pure PBS Buffer	-3.10
10 mM Alcohol in Buffer (Reference Solution)	-14.64
10 mM Glucose in Reference Solution	-15.07
10 mM Ascorbic Acid in Reference Solution	-14.88
10 mM Dopamine in Reference Solution	-14.73
10 mM Paracetamol in Reference Solution	-15.03
10 mM Amoxicillin in Reference Solution	-15.35
10 mM Estradiol in Reference Solution	-15.12

The standard deviation for all solutions, except pure PBS buffer, is 0.24 μA, which is insignificant compared with the 11.54 μA current change caused by the presence of 10 mM alcohol. Therefore, these physiological interferences do not affect the performance of the alcohol BC sensor, highlighting its excellent selectivity.

Table S3. Data statistics and analysis of chronoamperometric responses of BC alcohol sensors in artificial human sweat for alcohol concentrations of 0, 10, 20, 30 mM.

**Alcohol Concentration**	**0 mM** (Sample 1~5)	**10 mM** (Sample 6~10)	**20 mM** (Sample 11~15)	**20 mM** (Sample 16~20)
Chronoamperometric Current/ μA	4.57	6.46	9.32	13.02
Chronoamperometric Current/ μA	4.98	6.78	9.92	12.80
Chronoamperometric Current/ μA	4.78	7.14	9.58	12.31
Chronoamperometric Current/ μA	4.26	6.93	9.99	13.32
Chronoamperometric Current/ μA	5.35	7.45	10.22	13.38
**Average Value/ μA**	**4.79**	**6.95**	**9.80**	**12.96**
**Standard Deviation/ μA**	**0.369**	**0.335**	**0.390**	**0.390**

The limit of detection (LOD) is calculated based on the signal-to-noise ratio using the formula:

$$LOD=3\sigma/S$$

where σ represents the standard deviation of the background current measured in the absence of the target analyte, and S is the slope of the calibration curve. So σ here is 0.369 μA and S is 0.27 μA/mM, and the LOD is calculated as 4.1 mM.

The Nafion 117 solution contains approximately 5% Nafion polymer dissolved in a mixture of lower aliphatic alcohols and water, with a density of about 0.924 g/mL. The ethanol content is less than 4%. Additionally, only 2 μL of Nafion was applied to the electrode surface, corresponding to an ethanol concentration below 1.6 μM, which is negligible compared to our calculated limit of detection 4.1 mM. And the excellent linearity of the calibration curves for our alcohol BC sensors further supports this. Therefore, we believe that the trace amount of alcohol in the Nafion solution did not affect the performance of our sensors.

References

1. Baker, L. B., Physiology of sweat gland function: The roles of sweating and sweat composition in human health. *Temperature* **2019,** *6* (3), 211-259.
2. Hu, H.; Huang, H.; Li, M.; Gao, X.; Yin, L.; Qi, R.; Wu, R. S.; Chen, X.; Ma, Y.; Shi, K.; Li, C.; Maus, T. M.; Huang, B.; Lu, C.; Lin, M.; Zhou, S.; Lou, Z.; Gu, Y.; Chen, Y.; Lei, Y.; Wang, X.; Wang, R.; Yue, W.; Yang, X.; Bian, Y.; Mu, J.; Park, G.; Xiang, S.; Cai, S.; Corey, P. W.; Wang, J.; Xu, S., A wearable cardiac ultrasound imager. *Nature* **2023,** *613* (7945), 667-675.
